# A Transcriptional Regulatory Mechanism Finely Tunes the Firing of Type VI Secretion System in Response to Bacterial Enemies

**DOI:** 10.1128/mBio.00559-17

**Published:** 2017-08-22

**Authors:** Martina Lazzaro, Mario F. Feldman, Eleonora García Véscovi

**Affiliations:** a Instituto de Biología Molecular y Celular de Rosario, Consejo Nacional de Investigaciones Científicas y Tecnológicas, Universidad Nacional de Rosario, Rosario, Santa Fe, Argentina; b Department of Molecular Microbiology, Washington University School of Medicine, St. Louis, Missouri, USA; Massachusetts Institute of Technology

**Keywords:** *Serratia*, type VI secretion system, bacterial competition

## Abstract

The ability to detect and measure danger from an environmental signal is paramount for bacteria to respond accordingly, deploying strategies that halt or counteract potential cellular injury and maximize survival chances. Type VI secretion systems (T6SSs) are complex bacterial contractile nanomachines able to target toxic effectors into neighboring bacteria competing for the same colonization niche. Previous studies support the concept that either T6SSs are constitutively active or they fire effectors in response to various stimuli, such as high bacterial density, cell-cell contact, nutrient depletion, or components from dead sibling cells. For *Serratia marcescens*, it has been proposed that its T6SS is stochastically expressed, with no distinction between harmless or aggressive competitors. In contrast, we demonstrate that the Rcs regulatory system is responsible for finely tuning *Serratia* T6SS expression levels, behaving as a transcriptional rheostat. When confronted with harmless bacteria, basal T6SS expression levels suffice for *Serratia* to eliminate the competitor. A moderate T6SS upregulation is triggered when, according to the aggressor-prey ratio, an unbalanced interplay between homologous and heterologous effectors and immunity proteins takes place. Higher T6SS expression levels are achieved when *Serratia* is challenged by a contender like *Acinetobacter*, which indiscriminately fires heterologous effectors able to exert lethal cellular harm, threatening the survival of the *Serratia* population. We also demonstrate that *Serratia*’s RcsB-dependent T6SS regulatory mechanism responds not to general stress signals but to the action of specific effectors from competitors, displaying an exquisite strategy to weigh risks and keep the balance between energy expenditure and fitness costs.

## IMPORTANCE

*Serratia marcescens* is among the health-threatening pathogens categorized by the WHO as research priorities to develop alternative antimicrobial strategies, and it was also recently identified as one major component of the gut microbiome in familial Crohn disease dysbiosis. Type VI secretion systems (T6SSs) stand among the array of survival strategies that *Serratia* displays. They are contractile multiprotein complexes able to deliver toxic effectors directed to kill bacterial species sharing the same niche and, thus, competing for vital resources. Here, we show that *Serratia* is able to detect and measure the extent of damage generated through T6SS-delivered toxins from neighboring bacteria and responds by transcriptionally adjusting the expression level of its own T6SS machinery to counterattack the rival. This strategy allows *Serratia* to finely tune the production of costly T6SS devices to maximize the chances of successfully fighting against enemies and minimize energy investment. The knowledge of this novel mechanism provides insight to better understand bacterial interactions and design alternative treatments for polymicrobial infections.

## INTRODUCTION

*Serratia* is a Gram-negative genus that belongs to the family *Enterobacteriaceae* and encompasses species that can colonize a wide variety of environmental niches, ranging from water and soil to air. In addition to environmental ubiquity, among *Serratia* species, *S. marcescens* constitutes an emergent health-threatening nosocomial pathogen (1), with increasing reports of multidrug-resistant-strain outbreaks and high incidences in intensive and neonatal care units (2–4). *S. marcescens* has also recently been identified as one of the most abundant microbial species that colonizes the dysbiotic gut of Crohn patients, in detriment of beneficial bacteria (5). Furthermore, *S. marcescens* can interact either symbiotically or pathogenically with plants and insects. These features denote *S. marcescens*’ plasticity in adapting to changing ambient conditions and the deployment of strategies that allow the pathogen to colonize and thrive in complex polymicrobial niches.

Type VI secretion systems (T6SSs) have been found to be encoded in the genomes of a wide variety of Gram-negative bacteria, including both environmental and pathogenic microorganisms. These systems are assembled by a needlelike appendage that is able to export bacterial effectors and translocate them into an adjacent eukaryotic or prokaryotic cell. A T6SS assembles as a contractile device that displays homology to the tail spike complex of the T4 bacteriophage (6). Across different bacterial species, T6SSs differ in their capacity to liberate a variety of effectors, which are commonly associated with two conserved proteins encompassed within the estimated 13 core T6SS components: Hcp (hemolysin coregulated protein) a protein that forms the ejectable inner sheath of the nanotubular device, and VgrG (valine-glycine repeat G protein). VgrG trimmers form the spike of the needle, frequently associated with PAAR (proline, alanine, alanine, arginine) repeat motif-containing proteins at the tip of the spike (7). In order to prevent self or sister cell intoxication, T6SS^+^ organisms encode not only effectors but also immunity proteins to neutralize their cognate antibacterial effectors. Therefore, T6SSs are important components of the attack/defense force that bacteria deploy to antagonize other bacterial partners (intra- or intergenera) in order to survive and/or colonize a niche. In addition, some species rely on T6SSs to defeat the defenses of the invaded host (8–10).

While there is an increasing number of reports that shed light on detailed structural aspects of the apparatus assembly of the T6SS components and the mode of action of exported effectors across diverse bacterial species (11–13), the regulatory mechanisms that govern the expression of such complex and energetically costly devices are far from being understood.

The work of Iguchi et al. (14) provided evidences that the T6SS is part of the evolutionarily conserved weaponry of *S. marcescens*, dedicated to surmounting the challenge of surviving in a variety of environments. Nonetheless, in *Serratia*, the T6SS has been shown to be used in attacking bacterial competitors, and activity against eukaryotic targets has not been yet detected (15, 16). *S. marcescens* strain Db10 possesses a single T6SS with potent antibacterial activity, delivering at least six antibacterial effector proteins, including the peptidoglycan hydrolases Ssp1 and Ssp2 (15–18).

Previous work indicated that the expression of the T6SS in *S. marcescens* Db10 depends on one large transcriptional unit that encompasses the whole cluster and is constitutively active, as it was found to be independent of the growth phase, the growth medium composition, or contact with other bacterial cells assayed (16). PppA (phosphatase) and PpkA (kinase) proteins compose a reversible phosphorylation system that posttranslationally modifies Fha (forkhead associated), a component required for the assembly of the system (9). In some bacteria, such as *Pseudomonas aeruginosa*, this protein pair acts as part of a transduction cascade that functions as a posttranslational molecular switch to control T6SS assembly and activity upon detection of environmental and killed-sister-cell-derived signals (19, 20). However, although the PppA-PpkA switch controls the phosphorylation status of Fha in Db10, its activity was found to be at most modestly influenced by cell-cell contact, and it was postulated to finely adjust the bacterial killing capacity of the bacteria (21). More recently, a study by Gerc et al. (22) using single-cell techniques supported a model of a randomly triggered assembly of *S. marcescens* T6SS machinery, ready for attack on circumstantial prey.

Signal transduction systems and specialized secretory devices are key for bacteria to detect and adequately counteract the effects of noxious environmental conditions and defeat competitors. In our previous work, we have shown that in *S. marcescens* clinical isolate RM66262 (23), the Rcs system is key in controlling several traits of the bacterium. The Rcs signal transduction system is essentially composed of three proteins that belong to the two-component family: two inner membrane sensor proteins, RcsC (a bifunctional kinase/phosphatase sensor) and RcsD, and the cognate cytoplasmic transcriptional regulator RcsB. The phosphorylation status of RcsB modulates the binding of this regulator to promoter regions of the target genes, activating or repressing their transcription. Three additional components can be part of the Rcs-dependent signaling cascade: RcsF, a membrane-anchored lipoprotein that can channel stimuli to the RcsC sensor; IgaA, which is able to repress RcsC activity; and RcsA, which coregulates subsets of genes together with RcsB (24, 25).

Rcs system activity finely tunes the motile phenotypes (swimming and swarming) of *Serratia* by regulating the expression of FlhDC, the master regulator of the transcriptional flagellar cascade (26, 27). As a consequence, the Rcs system also modulates the capacity of *Serratia* to produce the PhlA phospholipase, which is transcriptionally regulated by FliA (the sigma factor that determines the secondary wave of the flagellar cascade expression) and exported through the flagellar secretome (26, 27). In addition, we have demonstrated that RcsB also controls the expression of *S. marcescens* ShlA, which belongs to the pore-forming toxin family of proteins (28). ShlA elicits an early autophagy response prior to *Serratia*’s internalization in nonphagocytic cells (28). Once *Serratia* cells have replicated inside a specialized vacuole, this effector triggers nonlytic egress of the bacteria from the infected cell by promoting an exocytic process (29). We have also shown that RcsB is involved in the regulation of *Serratia* outer membrane vesicle production (30). To summarize, we have demonstrated that the Rcs system is implicated in governing the expression of multiple phenotypes that are key to *Serratia*’s virulence potential.

In this work, we show for the first time that the T6SS expression levels of *S. marcescens* RM66262 are transcriptionally controlled by direct interaction of the RcsB response regulator with the promoter region of the T6SS gene cluster, a feature that appears to be highly conserved in *S. marcescens* strains, irrespective of the host or environmental source of the strain. We show that the RcsB-dependent modulation of the killing capacity of RM66262 against competitor bacteria is activated upon encountering rival bacteria. We reveal that RcsB-dependent induction of *Serratia* T6SS expression occurs in response to the detection of damage exerted by specific T6SS effectors translocated by competitors rather than to broad-spectrum envelope injury. Collectively, our results demonstrate that in *Serratia*, RcsB-controlled upregulation of the T6SS over basal expression levels constitutes a survival strategy triggered by specific lethal threats posed by interbacterial competition.

## RESULTS

### RcsB can alter *S. marcescens*’ interbacterial competition capacity.

**FIG 1:**
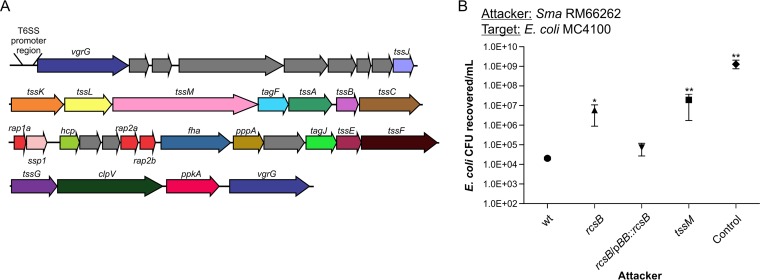
*S. marcescens* (*Sma*) kills *E. coli* MC4100 in a T6SS- and RcsB-dependent manner. (A) Schematic representation of the T6SS gene cluster of *S. marcescens* RM66262. (B) Recovery of viable *E. coli* MC4100 cells after coculture with the indicated *S. marcescens* RM66262 strains for 4 h at 37°C, with an initial 5:1 (attacker/target) ratio. *E. coli* DH5α mixed with *E. coli* MC4100 at a 5:1 ratio was used as the control. *S. marcescens* RM66262 *rcsB* and *tssM* mutant strains were 300-fold and 1,000-fold less proficient, respectively, than the wt strain in the ability to kill *E. coli*. The average values ± standard errors of the means (SEM) from four independent experiments are shown (*, *P* < 0.05; **, *P* < 0.01). wt, wild type.

Our bioinformatics analysis of the annotated genome of the *S. marcescens* clinical strain RM66262 (23) showed that it harbors a single canonical T6SS-encoding cluster, similar to those of previously analyzed *Serratia* strains (Fig. 1A) (14, 16). Because the Rcs signal transduction system regulates the expression of key virulence factors of *S. marcescens* (27, 28, 30), we examined whether this system affects the ability of *Serratia* to antagonize other bacteria. We carried out competition assays using RM66262 and *rcsB* and *tssM* mutants (unable to assemble the T6SS) derived from it as attacker strains and *Escherichia coli* MC4100, a strain naturally devoid of T6SS, as the prey. As shown by the results in Fig. 1B, *tssM* and *rcsB* strains were equally unable to kill the *E. coli* prey. This killing deficiency was restored to wild-type levels when the *rcsB* mutant was complemented in *trans* by the expression of RcsB from the pBB::*rcsB* plasmid. These results suggested that *S. marcescens*’ T6SS expression is under the control of RcsB.

### *Serratia marcescens*’ T6SS expression is under RcsB regulation.

**FIG 2:**
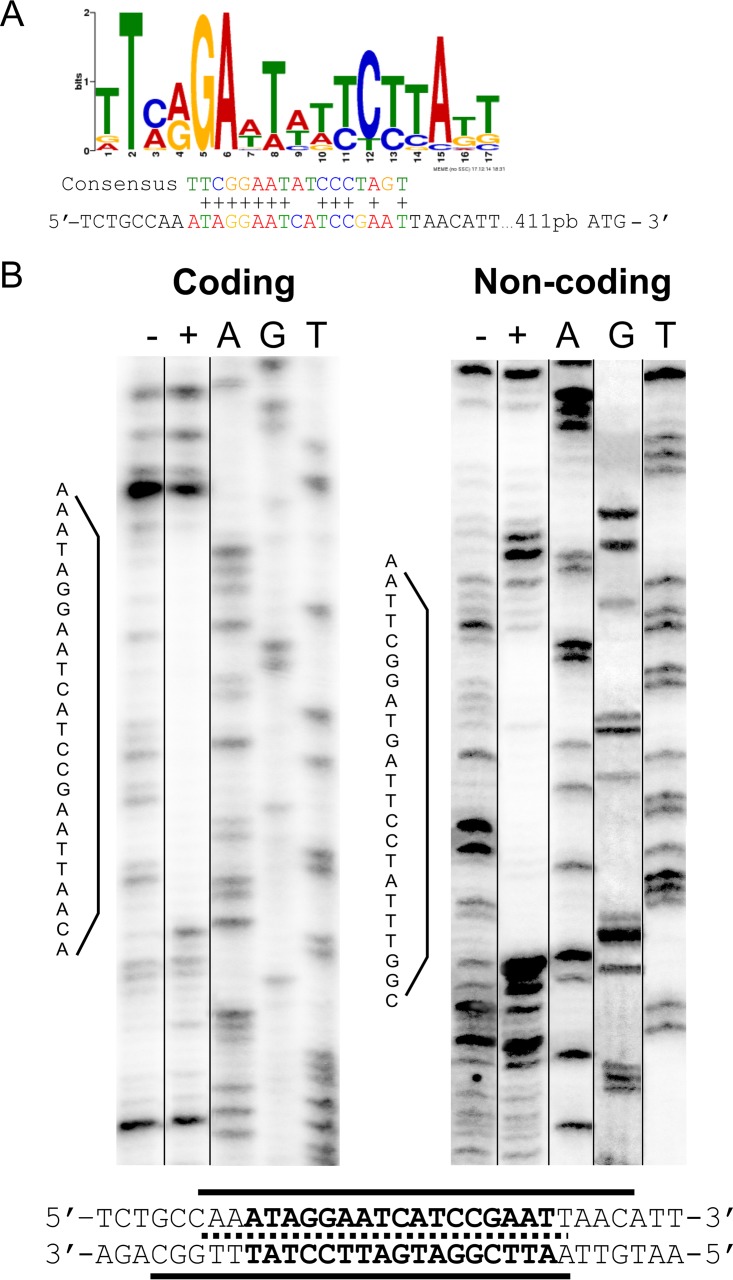
RcsB directly interacts with an RcsB-binding motif. (A) The logo shows the consensus motif for the RcsB-binding site, and the predicted RcsB-binding site sequence in the promoter region of *S. marcescens* RM66262 T6SS is indicated below the logo. The conserved residues are marked with “+” in the alignment with the consensus motif. (B) DNA footprinting analysis was performed on coding and noncoding strands of the T6SS promoter region. The DNA fragments were incubated with increasing amounts of purified RcsB. Results for 0 (−) and 25 (+) (the minimal protein concentration that showed protection) pmol of purified RcsB are shown. Ladders and the corresponding nucleotides are shown. The gels were sliced (noncontiguous lanes from a single gel are indicated by black lines) to exclude nonoptimal protein concentrations assayed or ladder lanes that resulted in smeared patterns. The nucleotide sequences of the RcsB-protected regions are indicated, and the protected DNA regions are underlined. The overlap of the protected sequences is marked with a dotted line.

We next performed an *in silico* search for a potential RcsB-binding motif upstream from *vgrG*, the first gene of the *S. marcescens* RM66262 T6SS cluster. By the use of the MEME/MAST motif detection programs (41, 42), we found the presence of a putative RcsB-binding motif upstream from *vgrG*’s ATG (Fig. 2A). This RcsB-binding sequence showed 12 of 17 conserved bases in the logo constructed by the search engine, using previously identified promoter regions that contain *bona fide* RcsB recognition sequences (Fig. 2A).

To assess whether there is direct interaction of RcsB with the *vgrG* promoter region and to better define the RcsB recognition sequence, DNase I protection assays were performed on both coding and noncoding strands. A nucleotide sequence encompassing 500 bp upstream from the *vgrG* ATG site and purified 6×His-tagged RcsB protein (RcsB-6×His) phosphorylated in the presence of acetyl phosphate were used, as described previously (28). We have previously corroborated that the 6×His tag does not alter either RcsB’s regulatory ability or its *in vitro* DNA-binding capacity (28). RcsB-6×His protected an overlapping region from nucleotide 418 to 438 relative to the *vgrG* translational ATG start site (Fig. 2B). This protected region encompassed the *in silico*-predicted RcsB-binding consensus motif.

An *in silico* analysis of upstream region sequences of the *vgrG* genes in available *Serratia* genomes deposited in the NCBI database was performed. As shown by the results in Fig. S1, a conserved motif with 100% sequence identity to that of RM66262 was found in the *Serratia* genomes that display synteny with the cluster borne by RM66262, in which *vgrG* was found to be the first gene of the T6SS cluster. This result is strongly indicative of conserved RcsB-dependent control of T6SS expression along the *Serratia* genus.

10.1128/mBio.00559-17.2 FIG S1  The RcsB-binding motif is conserved in the *Serratia* T6SS promoter region. Download FIG S1, PDF file, 2.3 MB. Copyright © 2017 Lazzaro et al. 2017 Lazzaro et al. This content is distributed under the terms of the Creative Commons Attribution 4.0 International license.

**FIG 3:**
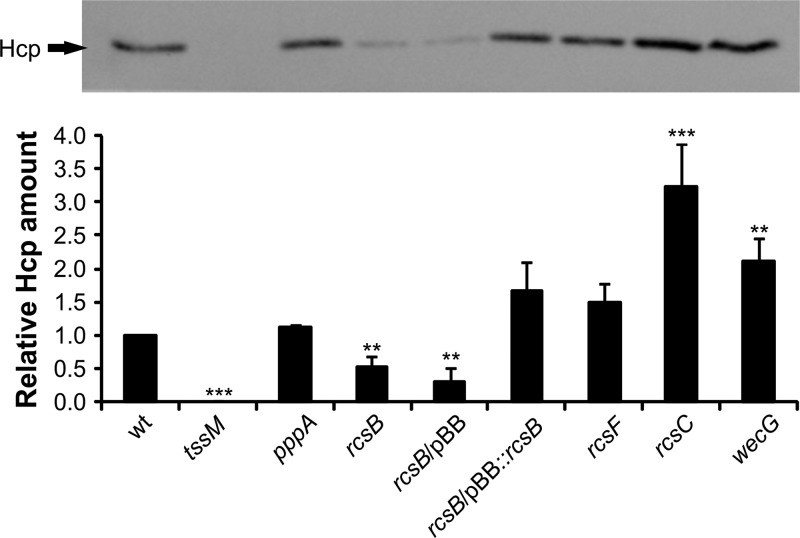
RcsB controls secretion of Hcp. Filtered supernatants from saturated cultures of the indicated *S. marcescens* RM66262 strains were precipitated and loaded into 15% SDS-PAGE gels. Hcp levels were determined by immunodetection using Hcp antisera followed by densitometry. (Top) Representative image of the assay. (Bottom) Average values ± standard deviations (SD) from three independent experiments are shown (**, *P* < 0.01; ***, *P* < 0.001). See Fig. S2 in the supplemental material for the densitometry normalization procedure employed.

Because Hcp composes the puncture device, which is expelled to the medium as it disassembles, the presence of Hcp in the culture supernatant is indicative of the dynamic assembly of the T6SS (33). To examine whether RcsB-dependent regulation is reflected at the level of protein expression, we determined the Hcp levels in strains with genetic backgrounds that alter Rcs function. Total culture supernatants from planktonic RM66262 wild-type (wt) or mutant strains grown in LB were trichloroacetic acid (TCA) precipitated and subjected to SDS-PAGE analysis and Coomassie blue staining or transferred to nitrocellulose and immunodetected with anti-Hcp polyclonal antibodies. Hcp expression levels were determined by densitometry (Fig. 3; Fig. S2). As predicted, Hcp levels were downregulated in the *rcsB* background and restored to wild-type levels when the *rcsB* strain was complemented in *trans* by the expression of RcsB from the pBB::*rcsB* plasmid (Fig. 3). Hcp was undetectable in the *tssM* mutant strain, while it was not affected by the *pppA* mutant background compared to its levels in the wild-type strain. In the *wecG* mutant strain, in which the Rcs system is activated (27), Hcp secretion was increased. *rcsF* inactivation did not alter Hcp secretion levels, while an *rcsC* background resulted in an increase in Hcp secretion levels (Fig. 3). These results were also in agreement with the results of reverse transcription-quantitative PCR (RT-qPCR) assays in which the transcriptional levels of *vgrG* (which codes for VgrG) and *hcp* (which codes for Hcp), distant from each other in the *Serratia* T6SS gene cluster, were analyzed in wild-type or *rcsB* planktonic bacteria grown in LB medium (Fig. S3A and B). To summarize, our results demonstrate that T6SS expression is modulated by RcsB at the transcriptional level in *S. marcescens* RM66262 and suggest that this regulatory mechanism might have been subjected to strong selective pressure along evolution in *Serratia*.

10.1128/mBio.00559-17.3 FIG S2  Hcp densitometry. Download FIG S2, PDF file, 0.8 MB. Copyright © 2017 Lazzaro et al. 2017 Lazzaro et al. This content is distributed under the terms of the Creative Commons Attribution 4.0 International license.

10.1128/mBio.00559-17.4 FIG S3  RcsB transcriptionally modulates T6SS expression. Download FIG S3, PDF file, 0.6 MB. Copyright © 2017 Lazzaro et al. 2017 Lazzaro et al. This content is distributed under the terms of the Creative Commons Attribution 4.0 International license.

### RcsB controls *S. marcescens* T6SS-mediated intraspecies competition.

**FIG 4:**
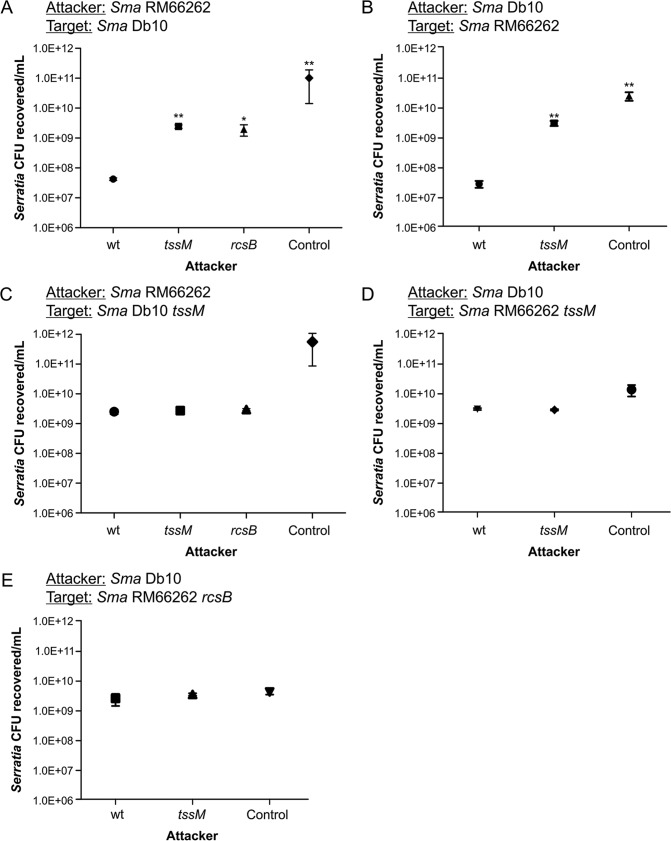
RcsB controls *Serratia* intraspecies T6SS-mediated competition. (A to E) Recovery of viable *S. marcescens* cells after 6 h of coculture with the indicated attacker strain at 37°C, with an initial ratio of 5:1 (attacker/target). *E. coli* DH5α mixed with *S. marcescens* at a 5:1 ratio was used for controls. *S. marcescens* RM66262 *tssM* or *S. marcescens* Db10 *tssM* mutants were 57-fold or 107-fold less proficient than their respective parental strains (wt) in outcompeting wild-type counterpart preys. *S. marcescens* RM66262 *rcsB* showed a 46-fold reduction in killing ability compared to that of the wild-type strain. Average values ± SEM from four independent experiments are shown (*, *P* < 0.05; **, *P* < 0.01).

*S. marcescens* T6SS has been described as functioning essentially in targeting competitor bacteria (21, 22, 34). Using the *S. marcescens* Db10 strain, previous reports have shown that the intraspecies killing of competing *Serratia* bacteria occurs irrespective of the functionality of the T6SS of the prey, discarding a counterattack, such as a “tit-for-tat” type of reciprocal response, for *Serratia* (22, 33). To evaluate the influence of RcsB-dependent regulation on the functionality of *Serratia* T6SS in intraspecies antagonism, we performed killing assays between *S. marcescens* strains Db10 and RM66262 and their *tssM* derivatives, as described in Materials and Methods. At a 5:1 attacker-to-prey ratio, both strains were able to kill their wild-type counterparts in a T6SS-dependent fashion, as demonstrated by the differences in the CFU counts of the prey recovered after attack by either the wild type or the *tssM* mutant (Fig. 4A and B). In these assays, the RM66262 *rcsB* strain showed a decrease in killing ability similar to that seen for the *tssM* strain compared to that of the wild-type strain (Fig. 4A).

However, neither the RM66262 nor the Db10 wild-type strain could kill its *tssM* counterpart strain in statistically significant numbers, and the same recovered CFU levels were obtained using either RM66262 *tssM* or Db10 *tssM* as the aggressor strain (Fig. 4C and D). Moreover, in the encounter with a *tssM* target strain, the results for an RM66262 *rcsB* attacker were also indistinguishable from the results for the wild-type or *tssM* attacker strain (Fig. 4C).

These results suggested that, in an intraspecific encounter, one *S. marcescens* strain will not attack another *S. marcescens* strain if the latter does not express an active T6SS. This is further supported by the observation that an *S. marcescens* RM66262 *rcsB* prey was not targeted by either a wild-type *S. marcescens* Db10 or a *tssM* attacker (Fig. 4E). This result also reinforces the involvement of RcsB in the control of *Serratia* T6SS expression.

### *S. marcescens* T6SS promoter activity is induced by kin T6SS^+^ competitors in an RcsB-dependent manner.

**FIG 5:**
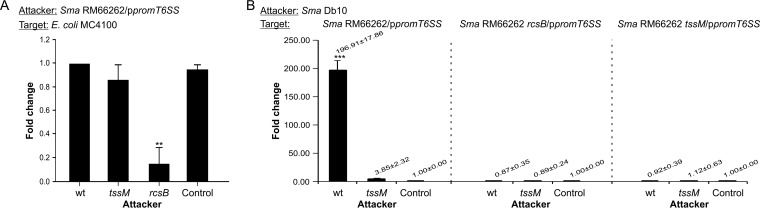
Intraspecies competition triggers *Serratia* T6SS activity in an RcsB-dependent manner. (A) *S. marcescens* RM66262/p*promT6SS* and the indicated mutant strains were incubated with *E. coli* MC4100 in a 5:1 (attacker/target) ratio for 4 h. *S. marcescens* RM66262/p*promT6SS* alone was used as the control. Serial dilutions were plated onto ampicillin-LB plates for *S. marcescens* RM66262 selection. GFP fluorescence/CFU was calculated. Fold change in transcriptional induction was calculated relative to wild-type strain values. Average values ± SD for four independent experiments are shown (**, *P* < 0.01). (B) *S. marcescens* Db10 wt or *tssM* mutant strains were incubated with *S. marcescens* RM66262 wt or mutant strains carrying the p*promT6SS* in a 5:1 (attacker/target) ratio for 6 h at 37°C. GFP fluorescence/CFU was determined. *E. coli* DH5α mixed with target bacteria at a 5:1 ratio was used for controls. Fold change in transcriptional induction was calculated relative to the control value. Average values ± SD from three independent experiments are shown (***, *P* < 0.001).

To analyze T6SS RcsB-dependent regulation upon exposure to T6SS-expressing (T6SS^+^) or T6SS-deficient (T6SS^−^) competitor strains, we cloned a 500-bp sequence upstream from *vgrG* (promoter) harboring the RcsB-binding motif in the pPROBE(NT') vector (31), resulting in the p*promT6SS* construct. This construct allows the quantitative detection of transcriptional activation by measuring the fluorescence of green fluorescent protein (GFP) expression driven by the upstream cloned sequence. As shown by the results in Fig. S3C, an expected *rcsB*-dependent response of the reporter was verified by introducing p*promT6SS* into the *S. marcescens* genetic backgrounds used in the Hcp secretion assays described above. In addition, we tested the p*promT6SS* response in an *S. marcescens*-versus-*E*. *coli* competition assay. Both wild-type and *tssM S. marcescens* strains showed *prom*T6SS expression levels that were equivalent to the value showed by the wild-type strain alone used as control. These basal expression levels depended on *rcsB* integrity (Fig. 5A).

Next, RM66262 strains harboring p*promT6SS* (prey) were challenged with either a Db10 wild-type or *tssM* strain (attackers). The fluorescence levels obtained from p*promT66S* contained in the RM66262 wild-type, *rcsB*, or *tssM* strain when coincubated with the control *E. coli* DH5α strain were measured. As shown by the results in Fig. 5B (left), the activity driven by the T6SS promoter increased ∼200-fold when the wild-type RM66262/p*promT6SS* strain was challenged by the wild-type Db10 strain. In contrast, the Db10 *tssM* strain was not able to induce T6SS promoter expression in RM66262/p*promT6SS*. Lack of RcsB expression resulted in the inability of the T6SS promoter to be induced when RM66262 *rcsB*/p*promT6SS* was challenged by either the wild-type or *tssM* Db10 strain (Fig. 5B, middle). In agreement with the incapacity of a wild-type strain to target either an *rcsB* or a *tssM* strain, the reporter was not induced from either the *rcsB* or the *tssM* background upon this encounter (Fig. 5B, right).

The latter results demonstrate that, in a *Serratia* intraspecific competition, the induction of a prey’s T6SS expression is RcsB dependent and occurs only when both attacker and prey carry a functional T6SS.

To further understand the *S. marcescens* competition strategy, we tested whether the stimulus detected by a T6SS^+^ *S. marcescens* strain in the encounter with a kin T6SS^+^ strain would be enough to enable the former strain to kill an otherwise undetected T6SS^−^ strain. As shown by the results in Fig. S4, both T6SS^+^ and T6SS^−^ preys are killed equally well when mixed with a T6SS^+^ attacker. Together, these results indicate that, as far as the expression of T6SS is triggered by the encounter with a threatening T6SS^+^ opponent, there is neither preferential nor directional aiming of a bystander prey, reinforcing the concept of a transcriptionally based response.

10.1128/mBio.00559-17.5 FIG S4  *Serratia*-induced T6SS can target preys in a nondirectional mode. Download FIG S4, PDF file, 1.8 MB. Copyright © 2017 Lazzaro et al. 2017 Lazzaro et al. This content is distributed under the terms of the Creative Commons Attribution 4.0 International license.

### Interspecies competition induces *Serratia* T6SS expression in an RcsB-dependent manner.

To further understand the signals that activate T6SS in *Serratia*, we employed competition assays between *Serratia* and *Acinetobacter baumannii* strain ATCC 17978. T6SS expression in *A. baumannii* ATCC 17978 is regulated by a plasmid that is spontaneously lost in part of the population. A strain without this plasmid possesses a constantly active T6SS (32). In all our experiments, we used either *A. baumannii* ATCC 17978 wild type or derivative strains lacking the repressing plasmid.

**FIG 6:**
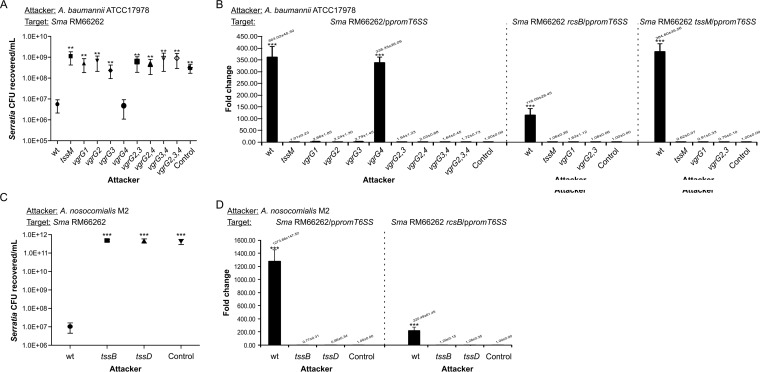
*Acinetobacter* killing capacity and RcsB-dependent induction of *Serratia* T6SS expression. (A, C) Recovery of viable *S. marcescens* RM66262 wt or mutant strains cocultured with wt or mutant strains of *A. baumannii* ATCC 17978 (A) or *A. nosocomialis* M2 (C) for 4 h at 37°C, with an initial 5:1 (attacker/target) ratio. *E. coli* DH5α mixed with target bacteria at a 5:1 ratio was used for controls. *A. baumannii* wt or *vgrG4* strains were 200-fold more proficient in outcompeting *Serratia* than the other *vgrG* single, double, or triple mutant strains analyzed. *A. nosocomialis* wt was ∼5 orders of magnitude more proficient in outcompeting *Serratia* than the mutant strains analyzed. Average values ± SEM from four independent experiments are shown (**, *P* < 0.01; ***, *P* < 0.001). (B, D) Wild-type or mutant strains of *A. baumannii* ATCC 17978 (B) or *A. nosocomialis* M2 (D) were coincubated with wt or mutant strains *S. marcescens* RM66262 carrying p*promT6SS* at an initial 5:1 (attacker/target) ratio for 4 h at 37°C. GFP fluorescence/CFU was calculated. *E. coli* DH5α mixed with target bacteria at a 5:1 ratio was used for controls. Fold change in transcriptional induction was calculated relative to the control value. Average values ± SD from four independent experiments are shown (***, *P* < 0.001).

We challenged *S. marcescens* RM66262 with either *A. baumannii* ATCC 17978 wild type or a *tssM* (T6SS^−^) strain in a 5:1 attacker-to-prey ratio. As shown by the results in Fig. 6A, the *A. baumannii* wild-type strain was able to kill *S. marcescens* RM66262 in a T6SS-dependent manner. In counterpart killing assays, also using a 5:1 attacker-to-prey ratio, the *Serratia* strain was unsuccessful at eliminating either T6SS^+^ or T6SS^−^ *A. baumannii* (Fig. S5A and B).

10.1128/mBio.00559-17.6 FIG S5  *Serratia*-*Acinetobacter* interspecific competition assays. Download FIG S5, PDF file, 1.5 MB. Copyright © 2017 Lazzaro et al. 2017 Lazzaro et al. This content is distributed under the terms of the Creative Commons Attribution 4.0 International license.

Challenge with wild-type *A. baumannii* caused an ∼400-fold induction of the expression of the *Serratia* T6SS promoter. This induction was dependent on the presence of an active T6SS system, as the *A. baumannii tssM* strain was not able to induce T6SS expression in *Serratia* (Fig. 6B, left, compare results from *A. baumannii* wild-type and *tssM* strains). To assess whether this induction depends on RcsB, the *S. marcescens* RM66262 *rcsB*/p*promT6SS* strain was also tested as prey. The abrogation of RcsB expression led to a 75% reduction of *prom*T6SS fold induction (Fig. 6B, middle), showing the dependence on RcsB for the activation of *Serratia*’s T6SS transcriptional activity upon encountering *Acinetobacter*. This activation was independent of the T6SS status of the prey, as both wild-type and *tssM S. marcescens* strains upregulated their T6SS as a consequence of the *Acinetobacter* attack. Our results show that *Acinetobacter* kills and also induces p*promT6SS* expression when coincubated with either a wild-type or *tssM S. marcescens* strain (Fig. 6A and B). Because the *Acinetobacter* strain used expresses its T6SS constitutively (32), the latter results indicate that, in this encounter, only *Serratia* follows a counterattack mode of action.

To verify that the phenotypes were not restricted to the interaction of *Serratia* with a unique *Acinetobacter* strain, we also determined that *Acinetobacter nosocomialis* strain M2 was able to kill *S. marcescens* RM66262, reducing the CFU count by 5 orders of magnitude (Fig. 6C). Only *A. nosocomialis* strain M2 carrying an active T6SS, and not isogenic *tssB* or *tssD* T6SS^−^ strains, was able to induce the transcriptional activity of RM66262 T6SS, by ∼1,300-fold in an RcsB-dependent fashion (Fig. 6D). It is important to highlight that a correlation between killing capacity and p*promT6SS* induction levels can be observed when the results from *A. baumannii* ATCC 17978 and *A. nosocomialis* M2 versus *S. marcescens* RM66262 are compared (Fig. 6A, C, B, and D, compare CFU values in killing assays and *prom*T6SS induction levels). Remarkably, if we also include *S. marcescens* intraspecies competition results in this comparative analysis (Fig. 4 and 5B), the correspondence between killing capacity and levels of *prom*T6SS upregulation can also be observed.

### Which signals are able to induce *Serratia* T6SS expression?

The components of *A. baumannii*’s T6SS have been comprehensively analyzed in a previous work by Weber et al. (13), showing that the T6SS gene cluster encodes four VgrG proteins with their associated putative effectors. VgrG1 has an essential role in Hcp export and, therefore, is a functional T6SS apparatus component. VgrG2 and VgrG3 would be required for the proper secretion of Tde and Tse effectors, respectively, which play a role in killing *E. coli* strains. Vgr4 and its cognate Tae effector were found to be dispensable for *E. coli* killing (13). As shown by the results in Fig. 6A, while Vgr4 was dispensable, VgrG2 and VgrG3 were simultaneously required for *A. baumannii* to kill *S. marcescens* RM66262. In correlation with this result, only *A. baumannii* wild-type and *vgrG4* strains were able to induce *prom*T6SS activity (Fig. 6B). Interestingly, the *vgrG2 vgrG3 vgrG4* triple mutant strain, despite proficiency in displaying a fully assembled T6SS (13), was inefficient in either killing *Serratia* or inducing *prom*T6SS activity (Fig. 6A and B). These results show that the simultaneous action of VgrG2 and VgrG3 cognate effectors is required both for killing and for RcsB-dependent T6SS induction. In agreement with the latter result, the sole inactivation of the Tse3 gene sufficed to make the wild type unable to kill *Serratia* or upregulate *S. marcescens* T6SS expression (Fig. S5C and D). Therefore, we ruled out the possibility that activation of the T6SS could simply be caused by a membrane perturbation provoked when *S. marcescens* encounters an *Acinetobacter* cell that carries a functional T6SS but is unable to deliver toxic effectors into *Serratia*. We then hypothesized that a component released by the killed *Serratia* subpopulation could act as a trigger of RcsB-dependent activation in the survivors. This hypothesis was supported by recent work in *Pseudomonas* indicating that kin cell lysate components are danger signals that can be detected by the prey (20).

Different approaches were designed to examine this possibility, as follows: (i) total lysate of wild-type *S. marcescens* RM66262 was incubated with RM66262/p*promT6SS*, and the transcriptional induction of the reporter was measured (Fig. S6A), and (ii), to more accurately obtain *S. marcescens* by-products released after a killing assay of *A. baumannii* against *Serratia*, the supernatant devoid of intact bacteria was collected and subsequently incubated with the reporter strain (Fig. S6B). In parallel, competition assays using *A. nosocomialis* M2 or *E. coli* DH5α (attackers) and wild-type *S. marcescens* RM66262 (prey) were carried out and a filter was placed on top where *S. marcescens* RM66262/p*promT6SS* strain was seeded, incubated for 4 h, and then collected to measure fluorescence (see Fig. S6C for a scheme of the approach). In addition, we performed a triple competition assay employing an attacker, a prey, and a reporter strain to determine whether self or nonself components released by dead cells *in situ* during the killing assay could induce T6SS expression. *S. marcescens* Db10 wild type or *tssM* (attacker) strains and *S. marcescens* RM66262 or *E. coli* MC4100 (prey) were coincubated with *S. marcescens* RM66262 *tssM*/p*promT6SS* (reporter) at a 5:1:1 ratio (Fig. S6D). None of these interactions induced the expression of the reporter, providing evidence that endogenous *Serratia* molecules released as the result of bacterial lysis or T6SS-provoked death are not able to trigger T6SS upregulation.

10.1128/mBio.00559-17.7 FIG S6  Bacterial lysis or derived products of T6SS-provoked killing are unable to activate *S. marcescens* T6SS expression. Download FIG S6, PDF file, 1 MB. Copyright © 2017 Lazzaro et al. 2017 Lazzaro et al. This content is distributed under the terms of the Creative Commons Attribution 4.0 International license.

Next, because the Rcs system is responsive to envelope stress, we also analyzed whether the actions of bacterial-envelope-damaging agents other than specific T6SS-dependent effectors were able to promote T6SS expression. To this end, and to exclude by-products of lethal action, the RM66262/p*promT6SS* reporter strain was incubated with sublethal concentrations of polymyxin B, deoxycholate, or SDS. To verify that each agent caused the expected perturbations of the bacterial envelope, their actions against the *Serratia* outer membrane were measured by β-lactamase (periplasmic) leakage and inner membrane disturbances by β-galactosidase (cytoplasmic) escape, using nitrocefin or *o*-nitrophenyl-β-d-galactopyranoside (ONPG) as the substrate, respectively (35). None of these challenges induced p*promT6SS* activity (Fig. S7A), although as expected, sublethal concentrations of each assayed agent were able to perturb bacterial membrane integrity (Fig. S7B to D).

10.1128/mBio.00559-17.8 FIG S7  Nonspecific envelope damage does not activate T6SS expression. Download FIG S7, PDF file, 1 MB. Copyright © 2017 Lazzaro et al. 2017 Lazzaro et al. This content is distributed under the terms of the Creative Commons Attribution 4.0 International license.

The latter results indicate that nonspecific envelope damage does not suffice to induce T6SS expression, strongly indicating that damage provoked by the specific action of T6SS effectors on *Serratia* cells generates the signal detected by the Rcs system that in turn upregulates T6SS expression.

## DISCUSSION

Interbacterial competition for a shared niche has evolutionarily selected refined mechanisms of defense and attack. T6SS is a potent weapon that endows bacteria with the ability to dynamically antagonize their competitors, turning kin and nonkin interactions into attacker-prey survival battles. To design new ways to fight polymicrobial infections, it is crucial to understand the dynamics of mixed bacterial populations that define the balance between species or determine the successful establishment and propagation of one dominant pathogen.

*S. marcescens*, as a typical opportunist and adaptable pathogen, is frequently isolated from polymicrobial infections in patients suffering from diseases such as pneumonia, meningitis, or urinary tract infections. Mixed bacterial populations include recalcitrant multiresistant *Acinetobacter* species, among other pathogens that can be *Serratia* cohabitants or contenders in the infected host (36–38).

In this work, we focused on understanding the mechanism that governs the firing of *S. marcescens*’ T6SS expression in response to intra- or interspecies encounters with rival bacteria. Our results show for the first time that in *S. marcescens*, the T6SS expression levels can be modulated at the transcriptional level. This regulation was found to be dependent on RcsB, the transcriptional regulator of the RcsCDB signal transduction system. We determined that RcsB is able to directly interact with a conserved RcsB-binding motif present in the promoter region of the T6SS gene cluster, governing the expression levels of both *vgrG* and *hcp*, as determined by quantitative RT-PCR assays. Taking into account previous work indicating that the *S. marcescens* T6SS gene cluster codes for one transcriptional unit (16), these results showed that RcsB controls *S. marcescens*’ T6SS expression. Detection of secreted Hcp in *Serratia* strains whose genetic backgrounds lead to the inhibition or activation of RcsB corroborated the idea that RcsB-dependent regulation affects the expression and assembly of *Serratia* T6SS. Interestingly, by *in silico* analysis, we found that the RcsB box motif has been highly conserved in the T6SS promoter region of a wide variety of *Serratia* strains, isolated either from hosts or ambient sources, indicating that a strong selective pressure might have evolutionarily preserved RcsB-dependent regulatory control over *Serratia* T6SS expression.

When *S. marcescens* strain RM66262 and its close relative *S. marcescens* strain Db10 were assayed in intraspecific competition experiments, an *rcsB* mutant strain displayed a killing deficiency similar to the one determined for a *tssM* mutant strain, which is unable to assemble the T6SS. This demonstrates that functional RcsB is necessary for *Serratia* to exhibit its killing ability against a kin competitor.

The results obtained while monitoring transcriptional expression from the RM66262 T6SS promoter during competition against Db10 clearly showed that T6SS expression is upregulated upon bacterial encounter. Furthermore, we demonstrated that this induction is RcsB dependent and occurs provided the two rival strains simultaneously hold a functional T6SS. These results reveal that, in *Serratia*-versus-*Serratia* antagonism, a strain activates the RcsB-dependent transcriptional expression and subsequent assembly of its T6SS weaponry when it detects signals of an aggressive T6SS^+^ opponent. It is tempting to associate this observed intraspecies behavior with the so-called “tit-for-tat” type of counterattack model originally described for *Pseudomonas* by Basler et al. (8). In that work, it was demonstrated that *Pseudomonas* T6SS assembly and counterpart killing were activated by signals elicited by the opponent’s T6SS apparatus, deploying the TagQRST/PppA-PpkA posttranslational signal/response cascade (it is pertinent to mention that *Serratia* has no TagQRST protein homologues encoded in its genome, and we found no T6SS transcription or Hcp secretion alterations in an *S. marcescens pppA* strain). However, we show herein that *S. marcescens* exhibits a transcriptional mode of competition strategy in which the signal elicited by the encounter with a T6SS^+^ contender enables the induced bacteria to target contiguous, otherwise harmless T6SS^−^ bacteria that share the same niche. In contrast to the tit-for-tat action, the *S. marcescens* strategy allows the menaced bacteria to display nonspatially oriented targeting of bystander preys.

Therefore, the competition-dependent *S. marcescens* T6SS firing behavior differs from that in previous reports postulating that, in *S. marcescens* Db10, the assembly of the T6SS is stochastic and ready for a circumstantial attack, irrespective of whether the encounter is with either aggressive T6SS^+^ or innocuous T6SS^−^ bacteria (22). These apparently contradictory results can be attributed to dissimilarities in the sets of genes encoding T6SS and, therefore, in the interplay among the particular array of effectors and immunity proteins expressed by each *S. marcescens* strain used for competition assays. Consequently, different *Serratia* strains would, in turn, generate distinct, specific signals for each encounter. We favor the hypothesis that *Serratia* expresses basal (or constitutive) T6SS levels, which depend on RcsB, and that these basal levels are sufficient to eliminate competitor bacteria devoid of T6SS effectors and immunity defenses, such as *E. coli* strain K-12-derived strains. This is based on the results shown in Fig. S3 in the supplemental material, demonstrating that T6SS expression in nonchallenged, planktonic *Serratia* cells is dependent on RcsB, and in Fig. 5A, showing that when *Serratia* cells encounter *E. coli* MC4100 cells, either a wild-type or a *tssM S. marcescens* strain displays RcsB-dependent T6SS expression levels that are equivalent to those obtained with an unchallenged *Serratia* strain.

We also determined that wild-type *Serratia* (either *S. marcescens* RM66262 or Db10) is not able to kill a *tssM* strain, a result that puts forth *Serratia*’s inability to detect and fire the T6SS machinery when an apparently harmless T6SS^−^ kin strain is encountered. In contrast to the results for *S. marcescens* RM66262 upon encountering *E. coli*, wild-type RM66262 or a *tssM* or *rcsB* mutant displayed an equivalent inability to kill a *S. marcescens* Db10 *tssM* mutant competitor, demonstrating that in an intraspecies encounter, only a T6SS^*+*^ prey is able to be detected by the aggressor and trigger T6SS upregulation in a RcsB-dependent manner. *Serratia* strains defective in T6SS assembly would, however, be protected against constitutive levels of T6SS effectors of its kin rival by the exposure of immunity proteins that specifically neutralize the lethal action of cognate effectors. In agreement with this result, both a *tssM* and an *rcsB Serratia* rival were equivalently unable to upregulate a wild-type *S. marcescens* strain’s expression of T6SS. Up to this moment, the comparison between *S. marcescens* RM66262 and Db10 genomes shows 12 proteins with amino acid sequence homology that are identified as T6SS effectors or immunity proteins (Fig. S8A). However, this is far from being an exhaustive analysis. Apart from those that do not have apparent counterparts, unassigned gene sequences that might code for T6SS effectors and/or immunity protein pairs or orphan ones in both strains could be found in the future. Taking into account that killing assays were carried out in a 5:1 attacker-to-prey relationship, we can conclude that upon encountering danger signals from *S. marcescens* T6SS^+^ opponents, even when partially sharing a similar set of effectors and immunity proteins, the expression of the T6SS system of a *Serratia* prey strain is transcriptionally upregulated over basal expression levels by RcsB in an attempt to counteract an enemy whose killing capacity outweighs the neutralizing ability of the prey.

10.1128/mBio.00559-17.9 FIG S8  (A) Schematic representation of the *in silico* comparison between *S. marcescens* Db10 and *S. marcescens* RM66262 gene clusters coding for T6SS effectors and immunity proteins. (B) Neither the PhoP/PhoQ nor the CpxRA system exerts a regulatory effect on T6SS expression. (C) Representative confocal z-slices of a competition assay between *A. nosocomialis* M2 and *S. marcescens* RM66262 wt/p*promT6SS*. Download FIG S8, PDF file, 1.3 MB. Copyright © 2017 Lazzaro et al. 2017 Lazzaro et al. This content is distributed under the terms of the Creative Commons Attribution 4.0 International license.

On the other hand, when we examined the interspecific interaction of *S. marcescens* with *Acinetobacter* strains, we found that either *A. baumannii* ATCC 17978 *or A. nosocomialis* M2 was able to efficiently kill *S. marcescens* strains. Strikingly, *Serratia* was unable to kill *Acinetobacter*, even when a 5:1 ratio was used, indicating a clear dominancy of *Acinetobacter* over *Serratia* under the conditions tested.

Previous results showed that in *A. baumannii*, a *vgrG1* mutant strain is impaired for Hcp secretion, while individual mutant strains with mutations in the other three *vgrG* genes (*vgrG2*, *vgrG3*, and *vgrG4*) do not display significant effects on Hcp secretion levels (13). As expected, an *A. baumannii vgrG1* strain was as attenuated as the *tssM* mutant strain in its capacity to kill *Serratia*. Moreover, while wild-type *A. baumannii* induced *S. marcescens*’ T6SS promoter transcription 400-fold, neither a *tssM* nor a *vgrG1* strain was able to upregulate its expression. A similar result was obtained when *A. baumannii vgrG2* and *vgrG3* strains were assayed. An estimated 100-fold induction was detected when a *Serratia rcsB* mutant was assayed as prey, suggesting that in this harsh encounter, an RcsB-independent, unknown regulatory mechanism is also put into play, partially contributing to the induction of *Serratia*’s T6SS expression.

The combinatorial effect of *vgrG2*, *vgrG3*, and *vgrG4* mutations indicated that Tde (a putative nuclease) and Tse (with unknown activity), the two cognate effectors whose secretion is facilitated by VgrG2 and VgrG3, respectively, were simultaneously required for killing and also for stimulating *S. marcescens*’ T6SS expression. Indeed, a mutant strain with a single Tse3 gene mutation was unable to either kill *S. marcescens* or upregulate its T6SS. Importantly, the fact that either double *vgrG2 vgrG3* or triple *vgrG2 vgrG3 vgrG4* mutant strains that fully assemble *A. baumannii* T6SS (13) were not able to upregulate the T6SS expression of *S. marcescens* allows us to rule out the idea that mere contact with an *Acinetobacter* T6SS apparatus that is assembled but incapable of translocating toxic effectors can constitute a signal for activating *S. marcescens* T6SS expression. It is worth emphasizing that *A. nosocomialis* strain M2 was found to be more aggressive than *A. baumannii* against *Serratia* and that a higher killing capacity correlated with an enhanced ability to activate *Serratia*’s T6SS expression, by up to 1,300-fold. This observation indicated that the extent of *Serratia*’s T6SS transcriptional induction is linked to the opponent’s capability of damaging the prey.

The RcsCDB phosphorelay is exclusively present in *Enterobacteriaceae* (24) and is known to act as a global regulatory network, controlling multiple cellular pathways, including capsule synthesis, cell division, motility, biofilm formation, and virulence mechanisms (25). The identity of specific signal(s) that activate the RcsCDB phosphorelay has remained elusive. However, a variety of environmental stimuli (high osmolarity and chlorpromazine treatment) and genetic mutations (*igaA*, *dsbA*, *rfa*, *wec*, *mdoH*, and *opg*) and the overexpression of certain proteins (DjlA, LolA, and OmpG) promote changes in the properties of the cell envelope, inducing the activity of the system in diverse bacterial species (25, 27). Nevertheless, we show that diverse agents, used in sublethal concentrations but causing measurable damage to either the outer (polymyxin B and sodium deoxycholate) or inner (SDS) membrane of *S. marcescens*, are not able to induce T6SS transcription in *S. marcescens*. This clearly indicates that cellular alterations generated by specific effectors are the source of the signal or signals able to trigger RcsB-mediated induction of *Serratia* T6SS promoter activity. In other enterobacteria, the Rcs and Cpx systems both respond to envelope stress, and their regulons partially overlap (39). As well, in *Edwardsiella tarda*, the PhoP/PhoQ system was shown to regulate T6SS expression (40). Our assays also indicate that neither the Cpx nor the PhoP/PhoQ system affects T6SS expression levels (Fig. S8B).

In *Pseudomonas aeruginosa*, LeRoux et al. (20) have shown that, upon killing by rival bacteria, the surviving subpopulation of *Pseudomonas* is able to detect its siblings’ lysis products by the Gac/Rsm signaling pathway and, in a posttranscriptional response, mounts the T6SS-dependent attack. Our assays showed that exposure of the T6SS reporter strain to *Serratia* cellular lysates, after-killing diffusible products, or cellular self components liberated *in situ* during the competition assay was not capable of triggering the T6SS promoter activity.

**FIG 7:**
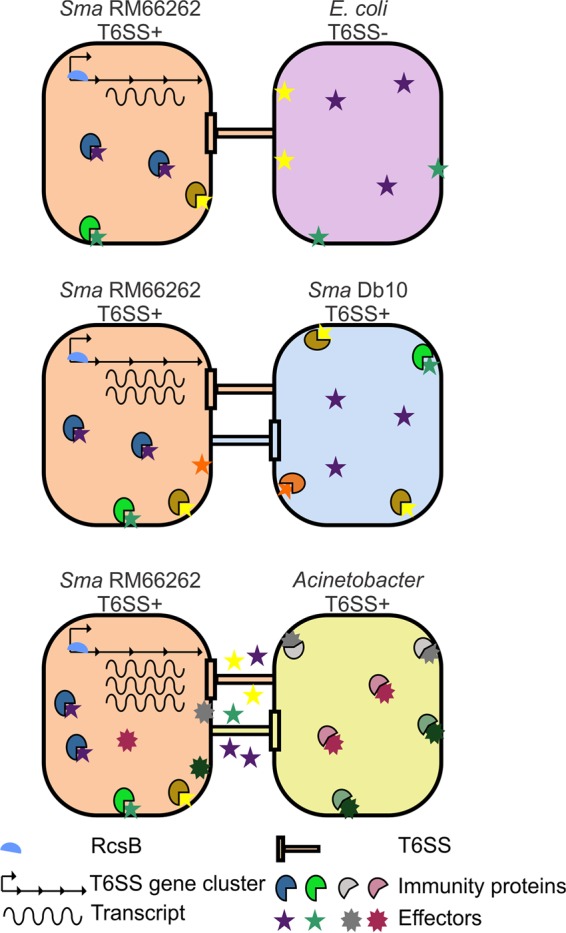
Schematic representation of the proposed model for the regulatory mechanism of the *S. marcescens* RM66262 T6SS (see Discussion for a full description).

Our results converge to demonstrate that, in *S. marcescens*, the specific action of either homologous or heterologous T6SS-derived effectors is recognized as a trigger signal and transduced to activate RcsB. In turn, activated RcsB interacts with a conserved recognition motif within the promoter region of T6SS and upregulates the expression of the killing machinery. As schematized in Fig. 7, we propose a model of action for the *S. marcescens* T6SS in which the extent of damage commands the response mechanism that is put into play. Challenge with *E. coli* MC4100, a natural T6SS^−^ strain that is efficiently killed by *Serratia*, was unable to induce the reporter’s transcriptional activity, indicating that a basal, uninduced level of T6SS expression is efficacious enough to get rid of defenseless rivals. When T6SS^+^ *S. marcescens* undergoes competition from kin T6SS^+^ *S. marcescens*, the extent of injury can be balanced by both shared offensive effectors and protective immunity proteins. This represents a moderate survival threat for the *Serratia* populations involved that would lead to one strain dominating, depending on the initial attacker-prey ratio. In this response, the cellular damage is weak and so is the RcsB-mediated induction of T6SS expression. A predator like *Acinetobacter* that does not discriminate T6SS^+^ or T6SS^−^ rivals exerts overwhelming cellular harm to *S. marcescens* in competition at a 5:1 ratio. The cellular damage inflicted by *Acinetobacter* under this condition cannot be countered by *Serratia* immunity proteins or halted by the killing action of *Serratia* effectors. Indeed, in a T6SS-deficient (*tssM*) *S. marcescens* strain, the RcsB-dependent induction is also triggered by an encounter with *Acinetobacter*. As a consequence, the RcsB-dependent response is amplified and T6SS expression is enhanced to high levels in an attempt to fight an antagonist that is a large-scale threat to the *Serratia* population’s survival. We predict that this response would be advantageous for *Serratia* in complex polymicrobial natural environments in which aggressor-prey ratios could be quite distinct from the conditions used in the experiments described herein. One nonlethal activating signal from a competitor would boost a *Serratia* survivor subpopulation with an improved capacity to fight other surrounding rivals.

Taken together, our results allow us to postulate that the detection-response system deployed by *Serratia* would enable this bacterium to weigh population damage versus energy expenditure at the time of expressing a complex machinery like the T6SS. In addition, the understanding of how this pathogen finely tunes the expression of its antibacterial armory will open new avenues for strategies that can be deployed to restrain *Serratia* infections.

## MATERIALS AND METHODS

### Bacterial strains, plasmids, and primers.

The strains, plasmids, and primers used in this study are listed in Table S1 in the supplemental material.

10.1128/mBio.00559-17.10 TABLE S1  Bacterial strains, plasmids, and primers used in this study. Download TABLE S1, DOCX file, 0.04 MB. Copyright © 2017 Lazzaro et al. 2017 Lazzaro et al. This content is distributed under the terms of the Creative Commons Attribution 4.0 International license.

### Antibacterial competition assays.

Competition assays were performed as described previously (16), with modifications as follows. Bacterial cells grown overnight were normalized to an optical density at 600 nm (OD_600_) of 0.5 and mixed at a 5:1 or 10:1 (attacker/target) ratio as indicated in each figure legend. Twenty-five microliters of this mixture was spotted onto a prewarmed agar plate and incubated at 37°C for 4 h or 6 h, as indicated. Cells were recovered from the spot and resuspended in 1 ml LB broth. Serial dilutions were plated out on antibiotic selection medium, using streptomycin for *E. coli* strain MC4100, tetracycline for *S. marcescens* strain Db10, ampicillin for *S. marcescens* strain RM66262, and chloramphenicol for *A. baumannii* strain ATCC 17978. Controls consisted of *E. coli* strain DH5α mixed with target bacteria at a 5:1 ratio. The recovery of viable cells is reported as the total number recovered per coculture spot. The results for each experiment are the average values of an assay performed in triplicate and independently repeated at least four times.

### Antibacterial competition assay with GFP transcriptional reporter.

The competition assays with a GFP transcriptional reporter were performed as described above, with modifications as follows. The target or attacker strain carried the p*promT6SS* construct as indicated. Controls consisted of *E. coli* DH5α mixed with target bacteria at a 5:1 ratio. Serial dilutions were plated out on kanamycin or ampicillin for selection of the reporter strain. Cells were washed three times with phosphate-buffered saline (PBS), and GFP fluorescence (excitation [λ_exc_] at 485 nm and emission [λ_em_] at 528 nm) was determined using a 96-microwell plate reader (Synergy2). Transcriptional induction was calculated as fluorescence divided by CFU. The fold change in transcriptional induction was calculated relative to the value obtained for the control or for the wild-type strain as indicated. We verified by simultaneous staining with propidium iodide that in competition assays, GFP fluorescence immediately extinguishes in dead bacteria and, therefore, GFP fluorescence detection corresponds only to live cells (see Fig. S8C and Text S1). The results for each experiment are the average values of an assay performed in triplicate and independently repeated at least three times.

10.1128/mBio.00559-17.1 TEXT S1  Supplemental methods are provided. Download TEXT S1, DOCX file, 0.1 MB. Copyright © 2017 Lazzaro et al. 2017 Lazzaro et al. This content is distributed under the terms of the Creative Commons Attribution 4.0 International license.

### Statistical analysis.

One-way analysis of variance (ANOVA) and the Tukey-Kramer multiple-comparison test with an overall significance level of 0.05 were used. In the figures, asterisks denote the values among the treatment groups in which a statistically significant difference was determined.
