# Supplementary material for: A Transcriptional Regulatory Mechanism Finely Tunes the Firing of Type VI Secretion System in Response to Bacterial Enemies
Source: mBio. 2017 Aug 22;8(4):e00559-17. doi: 10.1128/mBio.00559-17 (PMC5565961; doi:10.1128/mBio.00559-17)
Supplement: FIG S1 [file mbo004173445sf1.pdf]

***Serratia marcescens* RM66262**	CTGCCAA	ATAGGAATCATCCGAA	TTAACAT…	412 pb	ATG
***Serratia marcescens* subsp. *marcescens* Db11**	CCACCAA	ATAGGAATCATCCGAA	TTAACAT…	391 pb	ATG
***Serratia marcescens* SM39**	CTACCAA	ATAGGAATCATCCGAA	TTAACAT …	391 pb	ATG
***Serratia marcescens* CAV1492**	CCACCAA	ATAGGAATCATCCGAA	TTCACAT…	412 pb	ATG
***Serratia marcescens* B3R3**	CCACCAA	ATAGGAATCATCCGAA	TTAACAT…	412 pb	ATG
***Serratia marcescens* WW4**	CGGCCAA	ATAGGAATCATCCGAA	TTAACAT…	413 pb	ATG
***Serratia marcescens* RCS-14**	AGGCCAA	ATAGGAATCATCCGAA	TTAACAT…	413 pb	ATG
***Serratia marcescens* SmUNAM836**	CTGCCAA	ATAGGAATCATCCGAA	TTAACAT…	412 pb	ATG
***Serratia marcescens* U363635**	CCACCAA	ATAGGAATCATCCGAA	TTAACAT…	421 pb	ATG
***Serratia marcescens* isolate PWN146**	CCACCAA	ATAGGAATCATCCGAA	TTAACAT…	412 pb	ATG
***Serratia marcescens* SMB2099**	CCACCAA	ATAGGAATCATCCGAA	TTAACAT…	412 pb	ATG
***Serratia marcescens* AS1**	CCACCAA	ATAGGAATCATCCGAA	TTCACAT…	412 pb	ATG
***Serratia* sp. Fs14**	CGGCCAA	ATAGGAATCATCCGAA	TTAACAT…	413 pb	ATG
***Serratia* sp. SCBI**	CGGCCAA	ATAGGAATCATCCGAA	TTAACAT …	413 pb	ATG
