# Supplementary figures and images for: A Transcriptional Regulatory Mechanism Finely Tunes the Firing of Type VI Secretion System in Response to Bacterial Enemies
Source: mBio. 2017 Aug 22;8(4):e00559-17. doi: 10.1128/mBio.00559-17 (PMC5565961; doi:10.1128/mBio.00559-17)

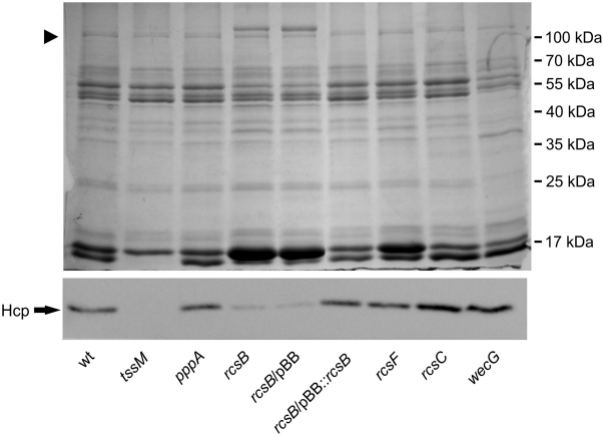

Supplement: FIG S2 [file mbo004173445sf2.pdf]

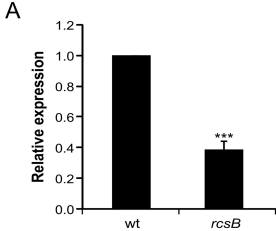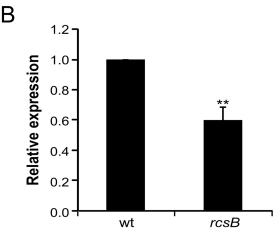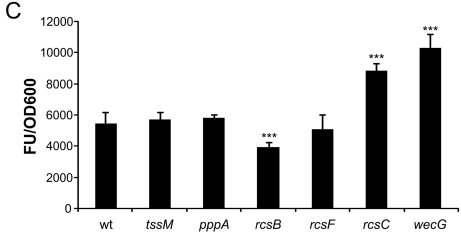

Supplement: FIG S3 [file mbo004173445sf3.pdf]

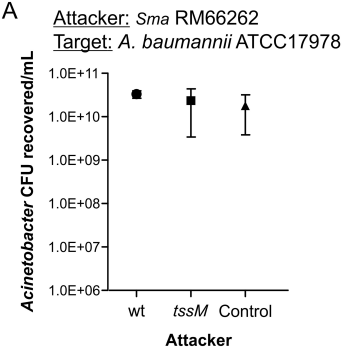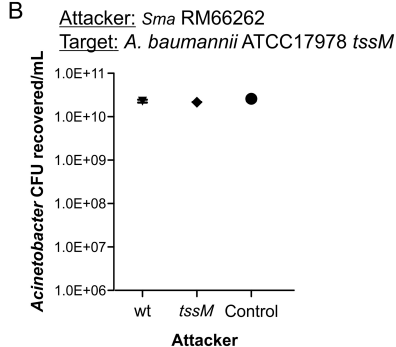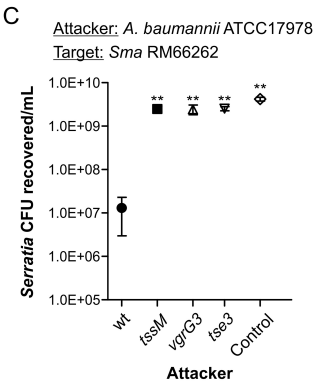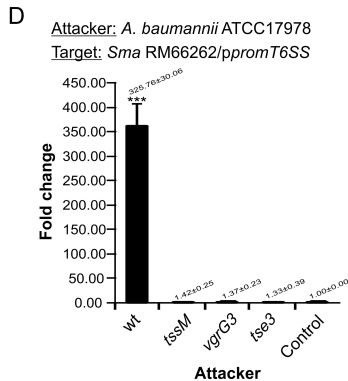

Supplement: FIG S5 [file mbo004173445sf5.pdf]

A

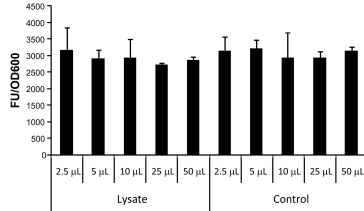

B

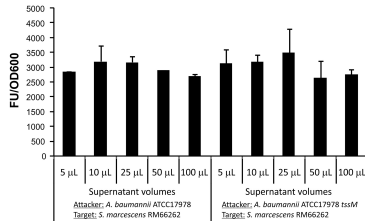

C

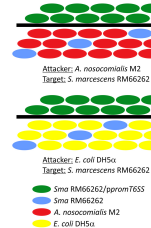

D

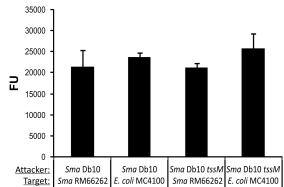

Supplement: FIG S6 [file mbo004173445sf6.pdf]

A

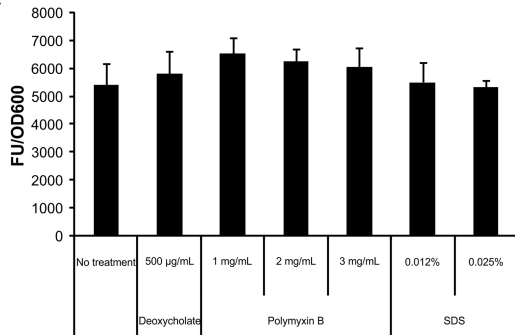

B

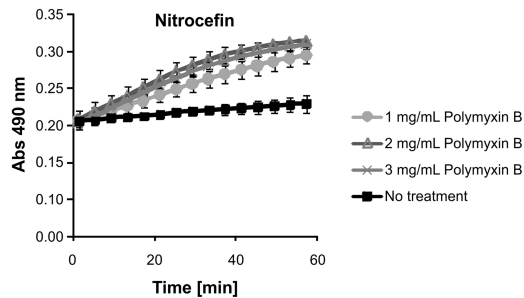

C

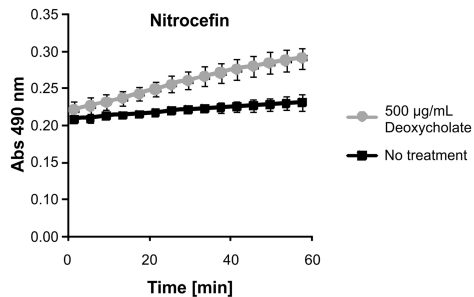

D

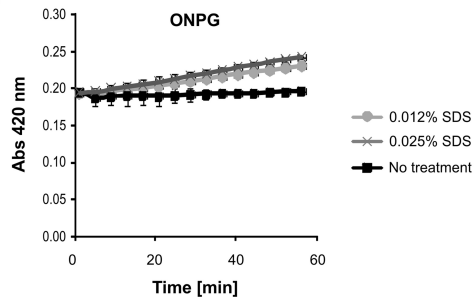

Supplement: FIG S7 [file mbo004173445sf7.pdf]

A

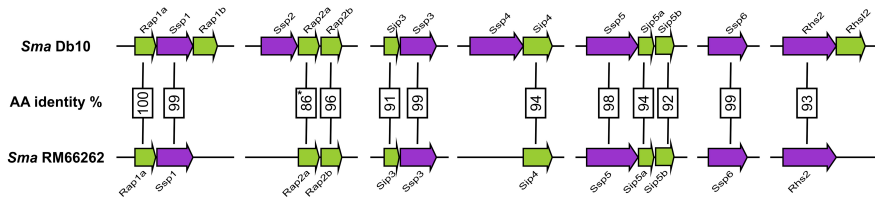

\* *Sma* RM66262 Rap2a lacks 11 AA N-terminal

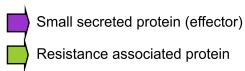

B

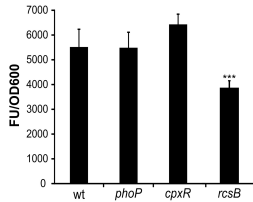

C

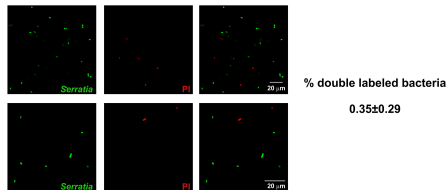

Supplement: FIG S8 [file mbo004173445sf8.pdf]
