# Supplementary material for: A Transcriptional Regulatory Mechanism Finely Tunes the Firing of Type VI Secretion System in Response to Bacterial Enemies
Source: mBio. 2017 Aug 22;8(4):e00559-17. doi: 10.1128/mBio.00559-17 (PMC5565961; doi:10.1128/mBio.00559-17)
Supplement: FIG S4 [file mbo004173445sf4.pdf]

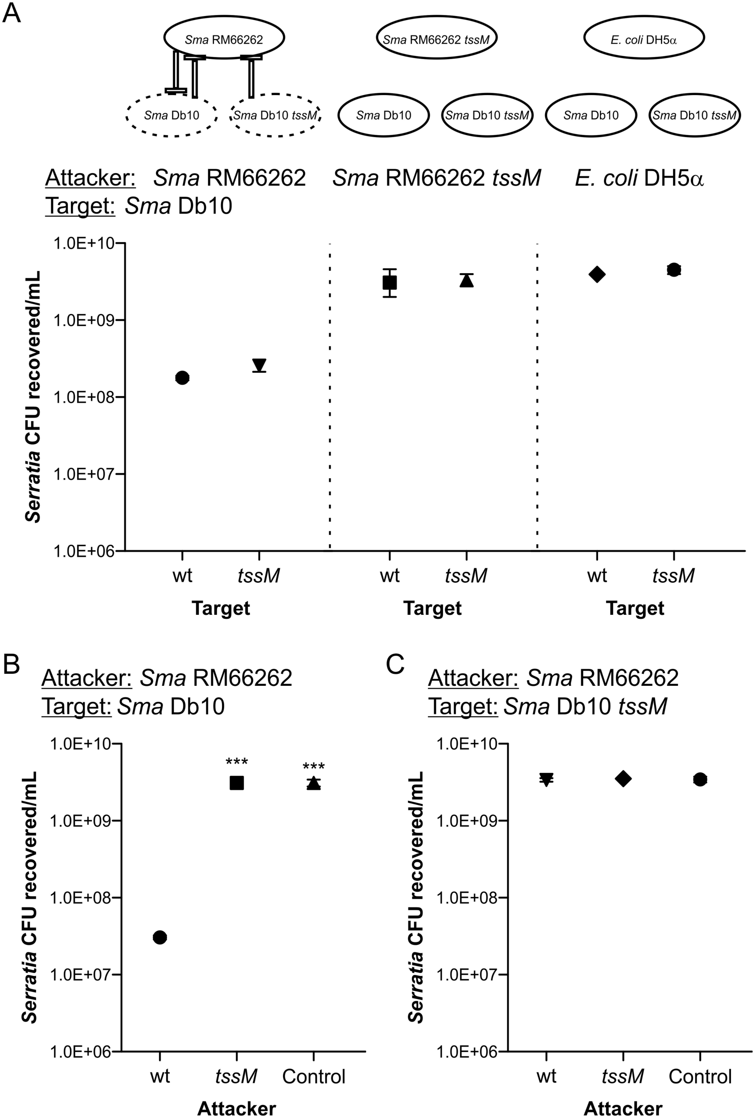
A
Sma RM66262
Sma RM66262 tssM
E. coli DH5α
Sma Db10
Sma Db10 tssM
Attacker: Sma RM66262 Sma RM66262 tssM E. coli DH5α
Target: Sma Db10
Serratia CFU recovered/mL
1.0E+10
1.0E+09
1.0E+08
1.0E+07
1.0E+06
wt
tssM
Target
B
Attacker: Sma RM66262
Target: Sma Db10
***
Control
Attacker
C
Attacker: Sma RM66262
Target: Sma Db10 tssM
