# Supplementary material for: A Transcriptional Regulatory Mechanism Finely Tunes the Firing of Type VI Secretion System in Response to Bacterial Enemies
Source: mBio. 2017 Aug 22;8(4):e00559-17. doi: 10.1128/mBio.00559-17 (PMC5565961; doi:10.1128/mBio.00559-17)
Supplement: TABLE S1 [file mbo004173445st1.docx]

**Table S1. Bacterial strains, plasmids and primers used in this study.**

| **Strains** | **Genotype and/or comments** | **Source or reference** |
| --- | --- | --- |
| ***S. marcescens* RM66262** | | |
| wild-type (wt) | *S. marcescens* RM66262; clinical isolate | ([1](#_ENREF_1)) |
| *tssM* | *tssM*::pKNOCK-Gm^R^ | This work |
| *pppA* | *pppA*::pKNOCK-Cm^R^ | This work |
| *rcsB* | *rcsB*::pKNOCK-Gm^R^ | ([2](#_ENREF_2)) |
| *rcsC* | *rcsC*::pKNOCK-Gm^R^ | ([2](#_ENREF_2)) |
| *rcsF* | *rcsF*::pKNOCK-Gm^R^ | ([2](#_ENREF_2)) |
| *wecG* | *wecG*::pKNOCK-Cm^R^ | ([2](#_ENREF_2)) |
| *phoP* | *phoP*::pKNOCK-Gm^R^ | ([3](#_ENREF_3)) |
| *cpxR* | *cpxR*::pKNOCK-Cm^R^ | This work |
| *wt/*pBB1 | Db10*/*pBBR1MCS | This work |
| *wt/*pBB2 | Db1*0/*pBBR1MCS-2 | This work |
| *tssM/*pBB1 | *tssM/*pBBR1MC*S* | This work |
| *tssM/*pBB2 | *tssM/*pBBR1MCS-2 | This work |
| *rcsB*/pBB | *rcsB*::pKNOCK-Gm^R^ /pBBR1MCS::*lacI* | ([4](#_ENREF_4)) |
| *rcsB*/pBB::*rcsB* | *rcsB*::pKNOCK-Gm^R^ /pBBR1MCS::*lacI*::*rcsB* | ([4](#_ENREF_4)) |
| wt/p*promT6SS* | *S. marcescens* RM66262 wt/ pPROBE(NT’)::*promT6SS* | This work |
| *tssM*/p*promT6SS* | *tssM*::pKNOCK-Gm^R^ / pPROBE(NT’)::*promT6SS* | This work |
| *pppA*/p*promT6SS* | *pppA*::pKNOCK-Cm^R^ / pPROBE(NT’)::*promT6SS* | This work |
| *rcsB*/p*promT6SS* | *rcsB*::pKNOCK-Gm^R^ / pPROBE(NT’)::*promT6SS* | This work |
| *rcsC*/p*promT6SS* | *rcsC*::pKNOCK-Gm^R^ / pPROBE(NT’)::*promT6SS* | This work |
| *rcsF*/p*promT6SS* | *rcsF*::pKNOCK-Gm^R^ / pPROBE(NT’)::*promT6SS* | This work |
| *wecG*/p*promT6SS* | *wecG*::pKNOCK-Cm^R^ / pPROBE(NT’)::*promT6SS* | This work |
| *phoP*/p*promT6SS* | *phoP*::pKNOCK-Gm^R^ / pPROBE(NT’)::*promT6SS* | This work |
| *cpxR*/p*promT6SS* | *cpxR*::pKNOCK-Cm^R^ / pPROBE(NT’)::*promT6SS* | This work |
| ***S. marcescens* Db10** | | |
| wild-type (wt) | *S. marcescens* Db10; non pigmented | ([5](#_ENREF_5)) |
| *tssM* | *tssM*::pKNG101-Sm^R^ | This work |
| ***A. nosocomialis* M2** | | |
| wild-type (wt) | *A. nosocomialis* M2, Metro Health Systems Clinical Isolate | ([6](#_ENREF_6)) |
| *tssB* | Strain M2 mutant containing an unmarked in-frame deletion of *tssB* | ([7](#_ENREF_7)) |
| *tssD* | Strain M2 mutant containing an unmarked in-frame deletion of *tssD* | ([7](#_ENREF_7)) |
| ***A. baumannii* ATCC17978** | | |
| wild-type T6+ (wt) | *A. baumannii* ATCC17978, T6SS active strain | ([8](#_ENREF_8)) |
| *tssM* | *A. baumannii* ATCC17978 T6+ Δ*tssM* | ([9](#_ENREF_9)) |
| *vgrG1* | *A. baumannii* ATCC17978 T6+ Δ*vgrG1* | ([9](#_ENREF_9)) |
| *vgrG2* | *A. baumannii* ATCC17978 T6+ Δ*vgrG2* | ([9](#_ENREF_9)) |
| *vgrG3* | *A. baumannii* ATCC17978 T6+ Δ*vgrG3* | ([9](#_ENREF_9)) |
| *vgrG4* | *A. baumannii* ATCC17978 T6+ Δ*vgrG4* | ([9](#_ENREF_9)) |
| *vgrG2,3* | *A. baumannii* ATCC17978 T6+ Δ*vgrG2,3* | ([9](#_ENREF_9)) |
| *vgrG2,4* | *A. baumannii* ATCC17978 T6+ Δ*vgrG2,4* | ([9](#_ENREF_9)) |
| *vgrG3,4* | *A. baumannii* ATCC17978 T6+ Δ*vgrG3,4* | ([9](#_ENREF_9)) |
| *vgrG2,3,4* | *A. baumannii* ATCC17978 T6+ Δ*vgrG2,3,4* | ([9](#_ENREF_9)) |
| *tse3* | *A. baumannii* ATCC17978 T6+ Δ*tse3* | This work |
| ***E. coli*** | | |
| MC4100 | Model laboratory *E. coli* K-12 strain; Sm^R^ (*rpsL150*) | ([10](#_ENREF_10)) |
| DH5α | General cloning strain | Laboratory Stock |
| **Plasmids** | | |
| p*promT6SS* | pPROBE(NT’)::*promT6SS* | This work |
| pBB1 | pBBR1MCS | ([11](#_ENREF_11)) |
| pBB2 | pBBR1MCS-2 | ([12](#_ENREF_12)) |
| pBB | pBBR1MCS::*lacI* | ([4](#_ENREF_4)) |
| pBB::*rcsB* | pBBR1MCS::*lacI*::*rcsB* | ([4](#_ENREF_4)) |
| pHis::*rcsB* | pHisParallel::*rcsB* | ([4](#_ENREF_4)) |
| pET::*hcp* | pET28a::*hcp* | This work |
| **Primers** | **Sequence (5´-3´)** | |
| TipoVI 1295 Fw 400 XbaI | TGCTCTAGAAGCCGTCAATATCTGTACC | |
| TipoVI 1295 Rv 800 XhoI | GACCTCGAGGTTCAATCAGGCGTTTGC | |
| 1308 tipo6 Fw 189 XbaI | TCCTCTAGAGCACCTGAACGCCCAG | |
| 1308 tipo6 Rv 570 XhoI | GACCTCGAGCTGGCTGAAGCTGTGC | |
| cpxR Fw XbaI | TCCTCTAGACCGGTCATCATGCTGCTGAC | |
| cpxR Rv XhoI | GACCTCGAGCGAGATGCTGCGCCAGC | |
| HcpFwNdeI | AAAGAGTGAAGAACATATGGCTATC | |
| HcpRvEcoRI | TACAAAGAATTCTTAAGCTTCGC | |
| prom SST6 Fw EcoRI | CGGAATTCGCTGCATTTTCAACCGTTCG | |
| prom SST6 Rv BamHI | TCGGATCCACTATTCCCCGCTTTAGGC | |
| vgrGRTFw | ATGGATCGCCTTATCATTG | |
| vgrGRTRv | CGCCTGCTTCCCGTCCGGAG | |
| hcpRTFw | ATGGCTATCGATATGTTCC | |
| hcpRTR | ACTCAACCTTGGTCAGGTG | |
| tse3KOFwd | TTAACAAAAAAACATCATCTTGACTTAAACCAAAATAATTCTGTCAGATGTGCGGTTAATTAAACCAAATCCTATAGAAACATATAATATAATTACACAGCGATTGTGTAGGCTGGAGCTGCTTCG | |
| tse3KORev | AAGATTTGAAACTGATGGCTCAGAACAAATTTCCATTCATATTTGTGATGAAAATGCTTCTAAATATAAACTAGCGGCAAAAGGTTAAAGATATAATGAATCATATGAATATCCTCCTTAGTTCCTATTCCG | |

Reference List

1. **Bruna RE, Revale S, Garcia Vescovi E, Mariscotti JF.** 2015. Draft Whole-Genome Sequence of Serratia marcescens Strain RM66262, Isolated from a Patient with a Urinary Tract Infection. Genome Announc **3**.

2. **Castelli ME, García Véscovi E.** 2011. The Rcs signal transduction pathway is triggered by enterobacterial common antigen structure alterations in Serratia marcescens. JBacteriol **193:**63-74.

3. **Barchiesi J, Castelli ME, Di Venanzio G, Colombo MI, Garcia Vescovi E.** 2012. The PhoP/PhoQ system and its role in Serratia marcescens pathogenesis. J Bacteriol **194:**2949-2961.

4. **Di Venanzio G, Stepanenko TM, Garcia Vescovi E.** 2014. Serratia marcescens ShlA pore-forming toxin is responsible for early induction of autophagy in host cells and is transcriptionally regulated by RcsB. Infect Immun **82:**3542-3554.

5. **Flyg C, Kenne K, Boman HG.** 1980. Insect pathogenic properties of Serratia marcescens: phage-resistant mutants with a decreased resistance to Cecropia immunity and a decreased virulence to Drosophila. J Gen Microbiol **120:**173-181.

6. **Niu C, Clemmer KM, Bonomo RA, Rather PN.** 2008. Isolation and characterization of an autoinducer synthase from Acinetobacter baumannii. J Bacteriol **190:**3386-3392.

7. **Carruthers MD, Nicholson PA, Tracy EN, Munson RS, Jr.** 2013. Acinetobacter baumannii utilizes a type VI secretion system for bacterial competition. PLoS One **8:**e59388.

8. **Basler M, Ho BT, Mekalanos JJ.** 2013. Tit-for-tat: type VI secretion system counterattack during bacterial cell-cell interactions. Cell **152:**884-894.

9. **Weber BS, Hennon SW, Wright MS, Scott NE, de Berardinis V, Foster LJ, Ayala JA, Adams MD, Feldman MF.** 2016. Genetic Dissection of the Type VI Secretion System in Acinetobacter and Identification of a Novel Peptidoglycan Hydrolase, TagX, Required for Its Biogenesis. MBio **7**.

10. **Casadaban MJ, Cohen SN.** 1979. Lactose genes fused to exogenous promoters in one step using a Mu-lac bacteriophage: in vivo probe for transcriptional control sequences. Proc Natl Acad Sci U S A **76:**4530-4533.

11. **Kovach ME, Phillips RW, Elzer PH, Roop RM, 2nd, Peterson KM.** 1994. pBBR1MCS: a broad-host-range cloning vector. Biotechniques **16:**800-802.

12. **Kovach ME, Elzer PH, Hill DS, Robertson GT, Farris MA, Roop RM, 2nd, Peterson KM.** 1995. Four new derivatives of the broad-host-range cloning vector pBBR1MCS, carrying different antibiotic-resistance cassettes. Gene **166:**175-176.
