# Supplementary material for: A Transcriptional Regulatory Mechanism Finely Tunes the Firing of Type VI Secretion System in Response to Bacterial Enemies
Source: mBio. 2017 Aug 22;8(4):e00559-17. doi: 10.1128/mBio.00559-17 (PMC5565961; doi:10.1128/mBio.00559-17)
Supplement: TEXT S1 [file mbo004173445s1.docx]

**Supplemental Material**

**Materials and Methods**

**Materials**. Antibiotics: kanamycin (50 µg/ml), chloramphenicol (20 µg/ml), streptomycin (100 µg/ml), tetracycline (12 µg/ml) and ampicillin (100 µg/ml), isopropyl-β-d-thiogalactopyranoside (IPTG; 0.5 mM), ortho-Nitrophenyl-β-galactoside (ONPG; 2 mM), sodium deoxycholate and sodium dodecyl sulfate (SDS) were provided by Sigma (Argentina). Polymyxin B sulfate was provided by Calbiochem. The secondary goat anti-rabbit IgG (H+L)-HRP conjugated was provided by Bio-Rad.

**Construction of insertion mutant** ***Sma* strains**. For *S. marcescens* RM66262, insertion mutations in *tssM,* *pppA* and *cpxR* were constructed with the pKNOCK suicide plasmid ([1](#_ENREF_1)). For each gene, an internal 400-bp region was amplified using primers TipoVI 1295 Fw 400 XbaI and TipoVI 1295 Rv 800 XhoI, 1308 tipo6 Fw 189 XbaI and 1308 tipo6 Rv 570 XhoI and cpxR Fw XbaI and cpxR Rv XhoI. The amplified products were digested with the restriction enzymes indicated in the primer names and cloned into the pKNOCK-Cm plasmid (*pppA* and *cpxR)* or pKNOCK-Gm plasmid (*tssM*). The resulting pKNOCK derivatives were conjugated into *S. marcescens* RM66262 and mutant strains were selected as chloramphenicol (Cm)- or gentamicin (Gm)-resistant colonies. As a result of homologous recombination between the cloned 400-bp internal region of each gene and the chromosomal copy of the gene, the 5’ region of the gene is separated from the 3’ region by insertion of the pKNOCK plasmid. The mutations were confirmed by PCR.

For *S. marcescens* Db10, insertion mutation in *tssM* was constructed with the pKNG101 suicide plasmid ([2](#_ENREF_2)). An internal 400-bp region was amplified using primers TipoVI 1295 Fw 400 XbaI and TipoVI 1295 Rv 800 XhoI. The amplified product was digested with the restriction enzymes indicated in the primer names and cloned into the pKNG101 plasmid. The plasmid was conjugated into *S. marcescens* Db10 and mutant strains were selected as streptomycin (Sm)-resistant colonies. As a result of homologous recombination between the cloned 400-bp internal region of each gene and the chromosomal copy of the gene, the 5’ region of the gene is separated from the 3’ region by insertion of the pKNG101 plasmid. The mutations were confirmed by PCR.

**Construction of *A. baumannii* *tse3* mutant strain**. T6+ *A. baumannii* ATCC 17978 Δ*tse3* was constructed by recombineering as described previously ([3](#_ENREF_3), [4](#_ENREF_4)). Briefly, an FRT-flanked kanamycin resistance cassette was amplified using with homology (100 bp) to the flanking regions of the targeted genes and homology (18-26 bp) to the 3′ region of the FRT sites flanking the Tn5 neomycin phosphotransferase gene from plasmid pKD4 ([5](#_ENREF_5)). The PCR product was purified and transformed into electrocompetent T6SS+ *A. baumannii* ATCC 17978 harboring pAT04 ([3](#_ENREF_3)), which encodes the RecAb recombinase. Mutants were selected on 7.5 μg/ml kanamycin, and integration of the resistance marker was confirmed by PCR. To remove the kanamycin resistance cassette, electrocompetent mutants were transformed with pAT03 ([3](#_ENREF_3)), which expresses the FLP recombinase. Mutants were confirmed by PCR and sequencing.

**Construction of p*promT6SS* plasmid.** The promoter region of the T6SS operon was amplified by PCR using the primers prom SST6 Fw EcoRI and prom SST6 Rv BamHI. The purified PCR product was digested with the EcoRI and BamHI restriction enzymes and was ligated into the same sites of pPROBE-NT’ ([6](#_ENREF_6)). The resulting plasmid was conjugated into *Sma* RM66262 wt strain, *tssM, pppA, rcsB, rcsC, rcsF, wecG, phoP* and *cpxR* mutant strains.

**Bacterial culture.** Bacteria were routinely grown in Millers Luria-Bertani (LB) medium supplemented with antibiotics or IPTG with good aeration, overnight at 37°C.

**RNA purification.** Total RNA was extracted from mid-exponential-phase cultures grown in LB medium at 37°C. 5 ml of ice-cold 5% water-saturated phenol (pH 5.5) in ethanol was added to 25 ml cultures to stop the degradation of RNA. Cells were centrifuged at 7000 g for 5 min at 4°C and resuspended in 5 ml of 10 mM Tris-HCl, 1 mM EDTA, pH 8.0. The RNA extraction was performed using the Promega SV Total RNA Isolation Kit, following the manufacturer’s instructions.

**Quantitative real-time RT-PCR.** cDNA synthesis was performed using random hexamers, 2 g of total RNA and 1 U of SuperScript II RNase H2 reverse transcriptase (Invitrogen). 5 µl of a 1/10 dilution of each cDNA was used as the template in quantitative real-time RT-PCR (reaction mixture, 20 μl), using primers vgrGRTFw and vgrGRTRv, and hcpRTFw and hcpRTRv. A primer set for the 16S rRNA was used as a control to confirm that equal amounts of total RNA were used in each reaction mixture. In every case the amplified fragment was of 250 bp. The reactions were carried out in the presence of the double-stranded DNA-specific dye SYBR green (Molecular Probes) and monitored in real time with a Mastercyclerep Realplex real-time PCR system (Eppendorf). The relative expression was calculated using the threshold cycle (CT) values obtained for each sample, as follows: 2^-ΔΔCT^, with ΔCT=CT_sample_-CT_16S_ and ΔΔCT=ΔCT_sample_-ΔCT_ref sample_, where ref sample is the reference sample, *Sma* RM66262. The average values were calculated from triplicate samples.

**DNase I footprinting assay.** DNase I protection assays were done with DNA fragments corresponding to the T6SS promoter region. DNA fragments were amplified by PCR using the appropriately ^32^P labeled primers (prom SST6 Fw EcoRI and prom SST6 Rv BamHI). Approximately 6.0 fmol of the DNA fragments were incubated with 25 pmol purified RcsB-Hisx6 protein at 30°C for 30 min, in a 20 μl volume of binding reaction mixture. RcsB-Hisx6 protein was previously phosphorylated by incubation with acetyl phosphate (0.9 ng/μl) at 30°C for 30 min. The binding buffer used for protein-DNA incubation contained 25 mM Tris-HCl (pH 8.0), 50 mM NaCl, 5 mM MgCl_2_, 5 mM dithiothreitol, 10% glycerol, 2.0 ng/µl salmon sperm DNA, and 0.025 mg/ml bovine serum albumin. DNase I (0.05 U; Life Technologies) was added and the mixture was incubated for 90 s at room temperature in a final volume of 100 µl. The reaction was stopped by adding 90 µl of 20 mM EDTA (pH 8.0) solution. DNA fragments were purified by phenol-chloroform extraction and resuspended in 6.0 µl of sequencing stop solution buffer (10 mM NaOH, 95% formamide, 0.05% bromophenol blue, 0.05% xylene cyanol). Samples were analyzed by denaturing polyacrylamide (6%) gel electrophoresis by comparison with a DNA sequence ladder generated with the appropriate primers.

**GFP transcriptional reporter assay.** Cultures were grown overnight in LB supplemented with kanamycin with good aeration at 37°C, and washed with PBS. GFP fluorescence (λ_exc_=485 nm, λ_em_=528 nm) and OD600 readings were determined with a 96-microwell plate reader (Synergy2). The fluorescence/OD600 (FU/OD600) ratio was calculated. The mean and standard deviations for triplicate analysis were calculated.

**Construction, expression and purification of Hisx6-tagged Hcp protein (Hcp-Hisx6).** A Hcp-Hisx6 tag fusion gene was constructed by PCR using primers HcpFwNdeI and HcpRvEcoRI and then cloned in the NdeI and EcoRI sites of pET28a vector. The Hisx6-tagged Hcp protein (Hcp-Hisx6) was expressed in *E. coli* BL21(DE3) pLysS. Cells were grown in 300 ml of LB at 37°C to an OD600 of 0.5. Following induction with 0.5 mM of IPTG and incubation for 4 h at 30°C, cells were harvested, resuspended in binding buffer (10 mM imidazole, 300 mM NaCl, 20 mM Tris-HCl, pH 8.0) and disrupted by sonication, followed by centrifugation. Supernatants were loaded onto a Ni^2+^-nitrilotriacetic acid-agarose affinity chromatography column equilibrated with 10 column volumes of binding buffer. The column was washed with 25 column volumes of washing buffer (20 mM imidazole, 300 mM NaCl, 20 mM Tris-HCl, pH 8.0). Bound protein was eluted using elution buffer (250 mM imidazole, 300 mM NaCl, 20 mM Tris-HCl, pH 8.0). Protein purity was determined by Coomassie stain following SDS-PAGE. Purified Hcp-Hisx6 protein was exhaustively dialyzed against PBS and protein concentration was determined by the bicinchoninic acid assay (Bio-Rad). Purified Hcp was used to immunize New Zealand rabbits, as described ([7](#_ENREF_7)).

**Determination of secreted Hcp protein levels in the by immunodetection.** The determination of protein levels by immunodetection was performed as described ([8](#_ENREF_8)). Briefly, 10 ml of cultures grown overnight with good aeration in LB at 37°C, and normalized by OD600. Cultures were centrifuged for 5 min at 5000 g and the supernatant was separated. The supernantant was filtered with 0.2 μm acetate-cellulose filters, precipitated with 12% trichloroacetic acid for 2 h at 4°C and centrifuged for 30 min at 30000 g. The precipitated secreted proteins were resuspended in protein sample buffer. 1 ml of trichloroacetic acid-precipitated spent medium was loaded onto 15% sodium dodecyl sulfate-polyacrylamide electrophoresis (SDS-PAGE) gels and transferred to Hybond-ECL nitrocellulose membranes. The membranes were blocked for 1 h with 5% non-fat milk, 0.1% Tris-Buffer Saline (TBS), and washed twice in TBS for 10 min. Then the blots were incubated with *S. marcescens* anti-Hcp rabbit polyclonal antibodies, washed twice in TBS and finally incubated with secondary antibody-HRP conjugated. The blots were developed using an enhanced chemiluminescence detection kit (Thermo Fisher Scientific). Quantification of individual bands by densitometry was performed using the Image J Program, using the Coomassie stained gel as load control. Relative Hcp amount was calculated relative to the value obtained for the *Sma* RM66262 strain. The assay was repeated three times.

**Diffusion experiments.** The assays were performed as previously described ([9](#_ENREF_9)) with modifications, as follows. Overnight cultures were washed, diluted to an OD600 of 0.5, mixed at a 5:1 attacker:target ratio and spotted onto LB agar plates. On top of the filter, the *Sma* RM66262/p*promT6SS* reporter strain (diluted to OD600 of 0.5) was spotted as depicted in Figure S6C. Following 4 h of incubation at 37°C, bacteria growing on top of the filter were resuspended in LB, washed with PBS and GFP fluorescence (λ_exc_=485 nm, λ_em_=528 nm) and OD600 readings were determined with a 96-microwell plate reader (Synergy2). The fluorescence/OD600 (FU/OD600) ratio was calculated. The results for each experiment are the average of an assay performed in duplicate and independently repeated three times.

**Lysate experiments.** Stationary phase *Sma* RM66262 was washed and resuspended in PBS before sonication. Different volumes of the lysate or PBS as control were mixed with *Sma* RM66262/p*promT6SS* reporter strain (diluted to OD600 of 0.5), and spotted onto a LB agar plate and incubated at 37°C for 4 h. Cells were recovered from the spot and resuspended in 1 ml LB and washed with PBS. GFP fluorescence (λ_exc_=485 nm, λ_em_=528 nm) and OD600 readings were determined with a 96-microwell plate reader (Synergy2). The fluorescence/OD600 (FU/OD600) ratio was calculated. The mean and standard deviations for triplicate analysis were calculated.

**Supernatant assay.** The assays were performed as the previously described antibacterial competence assay with modifications, as follows. After 4 h of incubation, cells were recovered from the spot, resuspended in 1 ml LB and supernatant was separated. The *Sma* RM66262/p*promT6SS* reporter strain (diluted to OD600 of 0.5) was mixed with different volumes of supernatant and spotted onto a LB agar plate. Following 4 h of incubation at 37°C, bacteria were resuspended in LB, washed with PBS and GFP fluorescence (λ_exc_=485 nm, λ_em_=528 nm) and OD600 readings were determined with a 96-microwell plate reader (Synergy2). The fluorescence/OD600 (FU/OD600) ratio was calculated. The results for each experiment are the average of an assay performed in duplicate and independently repeated three times.

**Tripartite bacterial competition assay.** The assays were performed as previously described bacterial competence assay with modifications, as follows. Bacterial cells grown overnight were normalized to an OD600 of 0.5, and mixed at a 10:1:1, attacker:target:target ratio. Then, 25 µl of this mixture was spotted onto a prewarmed agar plate and incubated for 6 h 37°C. Cells were recovered from the spot and resuspended in 1 ml LB. Serial dilutions were plated out on antibiotic selection: kanamycin for strains carrying the pBBR1MCS-2 plasmid or chloramphenicol for strains carrying the pBBR1MCS plasmid. Controls consisted of *E. coli* DH5α mixed with target bacteria at a 10:1:1 ratio. Target bacteria were conjugated with pBB1MCS or pBB1MCS-2 for antibiotic selection on chloramphenicol or kanamycin, respectively. The recovery of viable cells is reported as the total number recovered per cocultured spot. The results for each experiment are the average of an assay performed in triplicate and independently repeated three times**.**

**Tripartite bacterial competition assay with GFP transcriptional reporter.** The assays were performed as previously described bacterial competence assay with modifications, as follows. Bacterial cells grown overnight were normalized to an OD600 of 0.5, and mixed at a 5:1:1, attacker:target:reporter ratio. Then, 25 μl of this mixture was spotted onto a prewarmed agar plate and incubated for 6 h 37°C, as indicated. Cells were recovered from the spot, resuspended in 1 ml LB, washed with PBS and GFP fluorescence (λ_exc_=485 nm, λ_em_=528 nm) was determined with a 96-microwell plate reader (Synergy2). *Sma* RM66262 *tssM*/ppromT6SS was used as reporter in this assay. The results for each experiment are the average of an assay performed in triplicate and independently repeated three times.

**T6SS promoter region analysis****.** For genomic bioinformatics analysis, 13 complete genomic sequences of *Serratia* spp. (*S*. *marcescens* subsp. *marcescens* Db11 [HG326223.1], *S*. *marcescens* SM39 [[AP013063.1](https://www.ncbi.nlm.nih.gov/nucleotide/573008719?report=genbank&log$=nucltop&blast_rank=4&RID=APAR339B015)], *S. marcescens* CAV1492 [[CP011642.1](https://www.ncbi.nlm.nih.gov/nucleotide/828966150?report=genbank&log$=nucltop&blast_rank=9&RID=APAR339B015)], *S. marcescens* B3R3 [[CP013046.1](https://www.ncbi.nlm.nih.gov/nucleotide/943363765?report=genbank&log$=nucltop&blast_rank=5&RID=APAR339B015)], *S*. *marcescens* WW4 [[CP003959.1](https://www.ncbi.nlm.nih.gov/nucleotide/445210138?report=genbank&log$=nucltop&blast_rank=11&RID=APAR339B015)], *S. marcescens* RCS-14 [[CP012639.1](https://www.ncbi.nlm.nih.gov/nucleotide/926475601?report=genbank&log$=nucltop&blast_rank=12&RID=APAR339B015)], *S. marcescens* SmUNAM836 [CP012685.1], *S. marcescens* U363635 [[CP016032.1](https://www.ncbi.nlm.nih.gov/nucleotide/1039031667?report=genbank&log$=nucltop&blast_rank=6&RID=APAR339B015)], *S. marcescens* PWN146 [[LT575490.1](https://www.ncbi.nlm.nih.gov/nucleotide/1048493668?report=genbank&log$=nucltop&blast_rank=8&RID=APAR339B015)], *S. marcescens* SMB2099 [[HG738868.1](https://www.ncbi.nlm.nih.gov/nucleotide/1127886353?report=genbank&log$=nucltop&blast_rank=3&RID=APAR339B015)], *S. marcescens* AS1 [[CP010584.1](https://www.ncbi.nlm.nih.gov/nucleotide/1127556898?report=genbank&log$=nucltop&blast_rank=7&RID=APAR339B015)], *Serratia* sp. FS14 [[CP005927.1](https://www.ncbi.nlm.nih.gov/nucleotide/640856369?report=genbank&log$=nucltop&blast_rank=10&RID=APAR339B015)] and *Serratia* sp. SCBI [[CP003424.1](https://www.ncbi.nlm.nih.gov/nucleotide/676307183?report=genbank&log$=nucltop&blast_rank=13&RID=APAR339B015)]) were downloaded from NCBI.

The T6SS genes were identified using BLAST (https://blast.ncbi.nlm.nih.gov/Blast.cgi) to match *S*. *marcescens* RM66262 CDSs to all annotated T6SS genes from other *Serratia* spp. genomes. The strains that show synteny within the *Sma* RM66262 T6SS cluster were analyzed. The 500 bp upstream *vgrG* were considered as the T6SS promoter region and the RcsB-binding motif was searched in this region.

**Bacterial envelope stress assays.** *Sma* RM66262/p*promT6SS* cultures were grown overnight in LB supplemented with kanamycin and sub-lethal concentrations of SDS, sodium deoxycholate or polymyxin B sulfate, with good aeration at 37°C. Cultures were washed with PBS and GFP fluorescence (λ_exc_=485 nm, λ_em_=528 nm) and OD600 readings were determined with a 96-microwell plate reader (Synergy2). The fluorescence/OD600 (FU/OD600) ratio was calculated. Sub-lethal concentrations were determined for each agent: 0.025 % SDS, 500 µg/mL for deoxycholate and 3 mg/mL for polymyxin B. The mean and standard deviations for triplicate analysis were calculated.

**Outer membrane permeabilization assay.** The assay was performed as previously described ([10](#_ENREF_10)) with modifications, as follows. Bacteria were grown in LB supplemented with sodium dexycholate or polymyxin B sulfate, overnight at 37°C with good aeration. Cultures were diluted to OD600 of 0.6 in incubation buffer (10 mM phosphate buffer, pH 7.4, 100mM NaCl, containing 300 µg/ml LB) and added to a 96-microwell plate. Each well also contained 25 µL nitrocefin (120 µM) in phosphate buffer. Absorbance was followed at 490 nm for 60 min, with readings taken every 4 min at 37°C in a Synergy2 microplate reader, with shaking. The results for each experiment are the average of an assay performed in duplicate and independently repeated three times.

**Inner membrane permeabilization assays.** The assays were performed as previously described ([10](#_ENREF_10)) with modifications, as follows. Bacteria were grown in LB supplemented with SDS, overnight at 37°C with good aeration. Cultures were diluted to OD600 of 0.6 in incubation buffer (10 mM phosphate buffer, pH 7.4, 100mM NaCl, containing 300 µg/ml LB) and added to a 96-microwell plate. Each well also contained 25 µL ONPG (2 mM) in phosphate buffer. Absorbance was followed at 420 nm for 60 min, with readings taken every 4 min at 37°C in a Synergy2 microplate reader, with shaking. The results for each experiment are the average of an assay performed in duplicate and independently repeated three times.

**Verification of GFP fluorescence in live versus dead bacteria.** *A. nosocomialis* M2 wt was coincubated with *Sma* RM66262 wt/p*promT6SS*, for 4 h at 37°C, with an initial 5:1 (attacker:target) ratio. Cells were washed with PBS and each sample was stained with 3µM propidium iodide for 15 min at 37°C in the dark. At least 2000 GFP labeled bacteria were scored for double labeling detection. The samples were visualized with a Zeiss LSM880 confocal microscope and images were analyzed with ImageJ software. The mean and standard deviations for triplicate assays were calculated.

**Bioinformatics analysis.** To search for the putative RcsB-binding sites in the T6SS promoter region, the MEME/MAST tools were used. In brief, MEME is an algorithm which, given multiple input sequences, identifies one or more candidate motifs. MEME outputs the motif with the lowest (estimated) E value. Then, MAST searches for the motif in sequence databases using motifs with the position-specific probability matrix created by MEME ([11](#_ENREF_11), [12](#_ENREF_12)).

**Legends to Supplemental Figures**

**Fig. S1. The RcsB-binding motif is conserved in the *Serratia* T6SS promoter region*.*** An *in silico* analysis of the sequences upstream *vgrG* genes in available *Serratia* genomes deposited in the NCBI database was performed (accession numbers are provided in Supplementary Materials and Methods). The RcsB-binding conserved motif is underlined.

**Fig. S2. Hcp densitometry.** Filtered supernatants from saturated cultures of the indicated *Sma* RM66262 strains were precipitated and loaded into 15% SDS-PAGE gels. The protein band indicated with the arrow was used as load control for the densitometry shown in Fig. 3. The lower panel shows one representative Hcp immunodetection assay image.

**Fig. S3. RcsB transcriptionally modulates T6SS expression.** RT-qPCR analysis of (A) *vgrG* and (B) *hcp* expression. Total RNA was extracted from the *Sma* RM66262 wt or *rcsB* mutant strains grown to mid-exponential phase. *vgrG* and *hcp* expression was analyzed by RT-qPCR using specific primers (Table S1). 16S rRNA was used as internal control. Relative expression was normalized against the wt strain value. The average ± S.D. for three independent experiments is shown (** p< 0.01 and *** p< 0.001). (C) Cultures of the indicated *Sma* RM66262 strains carrying the p*promT6SS* were grown overnight. GFP fluorescence/OD600 (FU/OD600) was calculated. The average ± S.D. for three independent experiments is shown (*** p< 0.001).

**Fig. S4. *Serratia* induced T6SS can target preys in a non-directional mode** (A) Recovery of viable *Sma* Db10 wt and *tssM* mutant strain cocultured with *Sma* RM66262 wt or *tssM* mutant strain for 6 h, with an initial 10:1:1 (attacker:target:target) ratio. *E. coli* DH5α mixed with target bacteria at a 10:1:1 ratio was used as control. The upper schemes depict the bacterial interactions assayed. (B and C) Recovery of viable (B) *Sma* Db10 wt or (C) *tssM* mutant strain cocultured with *Sma* RM66262 wt or *tssM* mutant strain for 6 h, with an initial 10:1 (attacker:target) ratio. *E. coli* DH5α mixed with target bacteria at a 10:1 ratio was used as control. *Sma* RM66262 *tssM* was 107-fold less proficient than the wt strain in outcompeting *Sma* Db10 wt. The average ±SEM of three independent experiments is shown (*** p< 0.001).

**Fig. S5. *Serratia*-*Acinetobacter* inter-specific competence assays.** (A-B) Recovery of viable *A. baumannii* ATCC17978 (A) wt or (B) *tssM* mutant strains after 4 h of coculture with the indicated *Sma* RM66262 strain, with an initial 5:1 (attacker:target) ratio. *E. coli* DH5α and *A. baumannii* ATCC17978 at a 5:1 ratio were used as control. The average ± SEM for four independent experiments is shown. (C) Recovery of viable *Sma* RM66262 cocultured with *A. baumannii* ATCC17978 wt or mutant strains for 4 h, with an initial 5:1 (attacker:target) ratio. *E. coli* DH5α mixed with target bacteria at a 5:1 ratio was used as control. *A. baumannii* wt strains were 200-fold more proficient in out-competing *Serratia* than the other mutant strains analyzed. The average ± SEM for three independent experiments is shown (** p< 0.001). (D) *A. baumannii* ATCC17978 wt or mutant strains were coincubated with *Sma* RM66262 carrying p*promT6SS*, for 4 h, with an initial 5:1 (attacker:target) ratio. GFP fluorescence/CFU was calculated. *E. coli* DH5α mixed with target bacteria at a 5:1 ratio was used as control. Fold change in transcriptional induction was calculated relative to the control value. The average ± SD for three independent experiments is shown (*** p< 0.001).

**Fig. S6. Bacterial lysis or derived products of T6SS-provoked killing are unable to activate *Sma* T6SS expression.** (A) The indicated volumes of *Sma* RM66262 lysate or PBS as control were mixed with *Sma* RM66262/p*promT6SS* reporter strain and incubated for 4 h. GFP fluorescence/OD600 (FU/OD600) was calculated. The average ± SD for three independent experiments is shown. (B) *A. baumannii* ATCC17978 wt and *tssM* mutant strains were incubated with *Sma* RM66262 at a 5:1 (attacker:target) ratio for 4 h. Cells were recovered from the spot and the supernatant was separated. *Sma* RM66262/p*promT6SS* reporter strain was mixed with different volumes of the supernatant and spotted onto LB agar plates. After 4 h at 37°C, GFP fluorescence/OD600 (FU/OD600) was calculated. The average ± SD for three independent experiments is shown. (C) *A. nosocomialis* M2 or *E. coli* DH5α were incubated with *Sma* RM66262 in a 5:1 (attacker:target) ratio. *Sma* RM66262/p*promT6SS* reporter strain was spotted on top of the filter as depicted. Following 4 h of incubation at 37°C, bacteria grown on top of the filter were resuspended, GFP fluorescence/OD600 was calculated. (D) *Sma* Db10 wt or *tssM* mutant strains were incubated with *Sma* RM66262 or *E. coli* MC4100 and *Sma* RM66262 *tssM*/p*promT6SS* at a 5:1:1 (attacker:target:reporter) ratio for 6 h. Cells were washed with PBS and GFP fluorescence levels (FU) were determined. The average ± SD for three independent experiments is shown.

**Fig. S7. Nonspecific envelope damage does not activate T6SS expression.** (A) *Sma* RM66262/p*promT6SS* bacterial cultures supplemented with SDS, deoxycholate or polymyxin B at the indicated concentrations were grown overnight. GFP fluorescence/OD600 (FU/OD600) was calculated. The average ± SD for three independent experiments is shown. (B and C) *Sma* RM66262 strain was grown overnight, at 37°C, in LB supplemented with polymyxin B or deoxycholate, at the indicated concentrations. Cultures were diluted and nitrocefin was added. Readings of absorbance at 490 nm were taken every 4 min for 60 min. The average ± SD for three independent experiments is shown. (D) *Sma* RM66262 strain was grown overnight, in LB supplemented with SDS at the indicated concentrations. Cultures were diluted and ONPG was added. Readings of absorbance at 420 nm were taken every 4 min for 60 min. The average ± SD for three independent experiments is shown**.**

**Fig. S8. (A) Schematic representation of the *in silico* comparison between *Sma* Db10 and *Sma* RM66262 gene cluster coding for T6SS effectors and immunity proteins**. Green arrows represent ORFs encoding type VI-secreted proteins are and purple arrows represent associated immunity proteins. (B) **PhoP/PhoQ or CpxRA systems do not exert a regulatory effect on T6SS expression.** *Sma* RM66262 wt, *phoP,* *cpxR* or *rcsB* mutant strains were grown overnight. GFP fluorescence/OD600 (FU/OD600) was calculated. The average ± S.D. for three independent experiments is shown (*** p< 0.001).

(C) Representative confocal Z-slices of a competence assay between *A. nosocomialis* M2 (attacker) and *Sma* RM66262 wt/p*promT6SS* (prey; green fluorescence) are shown. After 4 hs of co-incubation, cells were stained with propidium iodide (red fluorescence). Bars: 20 µm. The average ± SD for three independent experiments is shown**.**

Reference List

1. **Alexeyev MF.** 1999. The pKNOCK series of broad-host-range mobilizable suicide vectors for gene knockout and targeted DNA insertion into the chromosome of gram-negative bacteria. Biotechniques **26:**824-826, 828.

2. **Kaniga K, Delor I, Cornelis GR.** 1991. A wide-host-range suicide vector for improving reverse genetics in gram-negative bacteria: inactivation of the blaA gene of Yersinia enterocolitica. Gene **109:**137-141.

3. **Tucker AT, Nowicki EM, Boll JM, Knauf GA, Burdis NC, Trent MS, Davies BW.** 2014. Defining gene-phenotype relationships in Acinetobacter baumannii through one-step chromosomal gene inactivation. MBio **5:**e01313-01314.

4. **Weber BS, Hennon SW, Wright MS, Scott NE, de Berardinis V, Foster LJ, Ayala JA, Adams MD, Feldman MF.** 2016. Genetic Dissection of the Type VI Secretion System in Acinetobacter and Identification of a Novel Peptidoglycan Hydrolase, TagX, Required for Its Biogenesis. MBio **7**.

5. **Datsenko KA, Wanner BL.** 2000. One-step inactivation of chromosomal genes in Escherichia coli K-12 using PCR products. Proc Natl Acad Sci U S A **97:**6640-6645.

6. **Miller WG, Leveau JH, Lindow SE.** 2000. Improved gfp and inaZ broad-host-range promoter-probe vectors. Mol Plant Microbe Interact **13:**1243-1250.

7. **Castelli ME, Fedrigo GV, Clementin AL, Ielmini MV, Feldman MF, Garcia Vescovi E.** 2008. Enterobacterial common antigen integrity is a checkpoint for flagellar biogenesis in Serratia marcescens. J Bacteriol **190:**213-220.

8. **Di Venanzio G, Stepanenko TM, Garcia Vescovi E.** 2014. Serratia marcescens ShlA pore-forming toxin is responsible for early induction of autophagy in host cells and is transcriptionally regulated by RcsB. Infect Immun **82:**3542-3554.

9. **LeRoux M, Kirkpatrick RL, Montauti EI, Tran BQ, Peterson SB, Harding BN, Whitney JC, Russell AB, Traxler B, Goo YA, Goodlett DR, Wiggins PA, Mougous JD.** 2015. Kin cell lysis is a danger signal that activates antibacterial pathways of Pseudomonas aeruginosa. Elife **4**.

10. **Arias M, Vogel HJ.** 2017. Fluorescence and Absorbance Spectroscopy Methods to Study Membrane Perturbations by Antimicrobial Host Defense Peptides. Methods Mol Biol **1548:**141-157.

11. **Bailey TL, Baker ME, Elkan CP.** 1997. An artificial intelligence approach to motif discovery in protein sequences: application to steriod dehydrogenases. J Steroid Biochem Mol Biol **62:**29-44.

12. **Bailey TL, Gribskov M.** 1998. Methods and statistics for combining motif match scores. J Comput Biol **5:**211-221.
